# Activation of Type I and III Interferon Signalling Pathways Occurs in Lung Epithelial Cells Infected with Low Pathogenic Avian Influenza Viruses

**DOI:** 10.1371/journal.pone.0033732

**Published:** 2012-03-21

**Authors:** Richard Sutejo, Dawn S. Yeo, Myint Zu Myaing, Chen Hui, Jiajia Xia, Debbie Ko, Peter C. F. Cheung, Boon-Huan Tan, Richard J. Sugrue

**Affiliations:** 1 Division of Molecular and Cell Biology, School of Biological Sciences, Nanyang Technological University, Singapore, Singapore; 2 Detection and Diagnostics Laboratory, DSO National Laboratories, Singapore, Singapore; McMaster University, Canada

## Abstract

The host response to the low pathogenic avian influenza (LPAI) H5N2, H5N3 and H9N2 viruses were examined in A549, MDCK, and CEF cells using a systems-based approach. The H5N2 and H5N3 viruses replicated efficiently in A549 and MDCK cells, while the H9N2 virus replicated least efficiently in these cell types. However, all LPAI viruses exhibited similar and higher replication efficiencies in CEF cells. A comparison of the host responses of these viruses and the H1N1/WSN virus and low passage pH1N1 clinical isolates was performed in A549 cells. The H9N2 and H5N2 virus subtypes exhibited a robust induction of Type I and Type III interferon (IFN) expression, sustained STAT1 activation from between 3 and 6 hpi, which correlated with large increases in IFN-stimulated gene (ISG) expression by 10 hpi. In contrast, cells infected with the pH1N1 or H1N1/WSN virus showed only small increases in Type III IFN signalling, low levels of ISG expression, and down-regulated expression of the IFN type I receptor. JNK activation and increased expression of the pro-apoptotic XAF1 protein was observed in A549 cells infected with all viruses except the H1N1/WSN virus, while MAPK p38 activation was only observed in cells infected with the pH1N1 and the H5 virus subtypes. No IFN expression and low ISG expression levels were generally observed in CEF cells infected with either AIV, while increased IFN and ISG expression was observed in response to the H1N1/WSN infection. These data suggest differences in the replication characteristics and antivirus signalling responses both among the different LPAI viruses, and between these viruses and the H1N1 viruses examined. These virus-specific differences in host cell signalling highlight the importance of examining the host response to avian influenza viruses that have not been extensively adapted to mammalian tissue culture.

## Introduction

Avian influenza viruses (AIV) are maintained in feral aquatic bird populations, which are thought to be the reservoir for the influenza A viruses that infect all other animal species [1]. Although AIV infection of domestic poultry is of economic importance, non-avian hosts, including humans can be infected [2], [3], [4]. Avian-to-human transmission of high pathogenic avian influenza (HPAI) viruses (e.g. H5N1) are often associated with high fatality rates, whereas associated fatalities due to human transmission of low pathogenic avian influenza (LPAI) viruses have not been reported. Poultry workers in China and Japan have tested seropositive for avian H5 and H9 suggesting prior infection [5], [6], and H9N2 infection in humans only results in mild influenza-like-illness [2]. In addition, AIVs can play a role in the evolution of seasonal influenza virus strains, with unpredictable consequences [7], [8]. Current AIV surveillance programs place a particular emphasis on H5 and H7 subtypes, since gradual introduction of mutations into the vRNA of LPAI viruses that are circulating in avian populations can lead to the emergence of HPAI viruses [9], [10], [11].

Pathogen-host interactions have been relatively well characterised in laboratory-adapted influenza viruses and in some HPAI virus isolates (e.g. H5N1), but in general our understanding of host interactions during AIV infection is comparatively poor. Although current animal model systems can provide useful information about the pathology of specific influenza virus isolates, they (e.g. mice) are not naturally infected with influenza viruses, and they respond to the virus infection in an age-dependant manner [12], [13]. In general these viruses need to be adapted to their new host, and during the process of species adaptation inherent biological properties of these viruses can be lost or modified. Cell culture systems that are permissive for LPAI virus infection can provide an additional useful complementary experimental approach to analyse the fundamental biological properties of non-mammalian adapted LPAI virus isolates that would otherwise grow poorly in mammalian hosts. Many of these permissive cell types (e.g. A549) retain complete signalling networks that are related to the innate host response to infection [e.g. interferon (IFN)], and this can be used to examine the host response to AIV infection. Furthermore, it is expected that these cell types retain elements of these signalling networks that are species specific, i.e. they retain biological properties of the species from which they are derived. Additionally, because virus infection of cell culture systems can be accurately controlled, specific molecular and cellular changes (e.g. host gene expression) in the host cell that occur early in the course of infection can be analysed.

The capacity of HPAI viruses to cause high fatality rates in humans is not shared by most other AIVs, and the majority of circulating AIVs are LPAI viruses. The host response to virus infection plays a pivotal role in the disease progression and several studies have described a systems biology approach to examine the host response in influenza virus causing disease in humans. Although such approaches have been used to examine the host response to AIV infection, this has been restricted to HPAI viruses such as the H5N1 virus [14], and similar analyses has not been performed on circulating LPAI viruses. An improved understanding of the host response to representative circulating LPAI viruses should enhance our general understanding of the biological properties of these AIVs, and may additionally improve our understanding of the relevance of the host responses to HPAI viruses.

In this report we describe a systems approach to examine the host responses of representative LPAI viruses that were circulating in SE Asia. These viruses were isolated from live broiler ducks imported into Singapore during routine surveillance. These viruses, were the first LPAI viruses isolated in South-East Asia that were completely characterised at the genetic level, and include H5N2, H5N3 and H9N2 virus subtypes [15]. This genetic analysis indicated that they contained avian amino signature sequence motifs in all virus proteins, consistent with their avian host specificity. In this study we provide the first comprehensive analysis of the replication characteristics of these viruses, and their effect on the host cell transcriptome in different permissive cell types of mammalian and avian origin. The properties of these viruses were compared with that of the laboratory-adapted human H1N1/WSN isolate, and several pH1N1 viruses that were isolated from humans in Singapore during the influenza pandemic in 2009 [16]. We show that although all viruses examined could replicate in each of the cell types examined, a significant difference in the host response between the AIVs and the human virus isolates within each cell type was observed. In human cells the replication of the AIVs correlated with a robust activation of Type 1and Type III IFN and cell-death signalling pathway, while a reduced interferon response was observed in cells infected with the H1N1 viruses. In CEF cells activation of IFN signalling pathways was not observed following AIV infection, while increased IFNβ was observed in H1N1/WSN infected cells. These data suggest virus-specific differences in the replication characteristics and host responses of these LPAIs, both when compared with the human viruses examined in this study, and when compared with previous observations that have been reported for HPAI viruses.

## Results and Discussion

### Replication characteristics of the viruses used in this study in A549, CEF and MDCK cells

**Figure 1:**
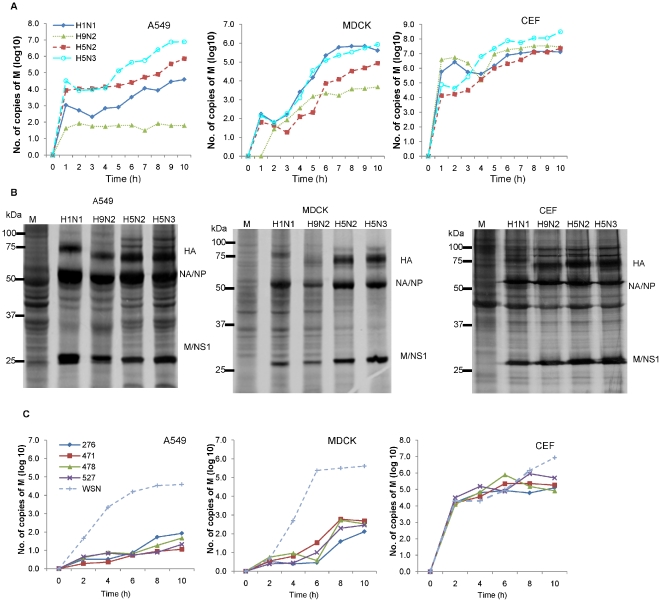
Replication properties of the AIV and pH1N1 viruses in mammalian and avian cell types. A549, MDCK, and CEF cells were infected either with the H1N1/WSN (♦), H9N2 (▴), H5N2/F118 (▪) or H5N3 (○) viruses using an MOI = 4 and incubated at 37°C. (A) At hourly intervals post infection the cells were harvested and the vRNA levels quantified using qPCR as described in methods. Each value at a specific time point represents the mean of triplicate measurements (p<0.05). The data presented are a representative data set from one of two independent experiments. (B) At 9 hpi mock (M) infected cells or cells infected with either of the four viruses were radiolabelled for 1 hr in DMEM minus methionine (Invitrogen, USA) containing 100 µCi/ml [^35^S] methionine (Perkin-Elmer, USA). Cells were extracted in boiling mix and analysed by SDS-PAGE. Protein bands corresponding to the neuraminidase protein (NA)/nucleoprotein (NP), the matrix (M) protein and nonstructural 1 (NS1) protein, and the uncleaved heamaggultinin (HA) are indicated. (C) Cells were infected with either the pH1N1/276 (♦), pH1N1/471 (▪), pH1N1/478 (▴), pH1N1/527(X) or H1N1/WSN (+) viruses, and at hourly intervals post infection the cells were harvested and the vRNA levels quantified using qPCR as described in methods. Each value at a specific time point represents the mean of triplicate measurements (p<0.05). The data presented are a representative data set from one of two independent experiments.

Prior to examination of the host response to infection we examined the biological properties of the H5N2/F118, H5N2/F189, H5N2/F59, H5N3 and H9N2 viruses used in this study in the different cell types. In all cases and unless otherwise specified, a multiplicity of infection (MOI) of 4 was used throughout this study. The replication kinetics was established for the H1N1/WSN, H5N2/F118, H5N3 and H9N2 viruses in each cell type by performing RNA quantification at 1 hr intervals up to 10 hpi (Figure 1A). The M gene universal diagnostic primer was used in quantitative PCR (qPCR) analysis to measure the vRNA levels [17], since we found that segment 7 kinetics was representative of the general trend in vRNA synthesis [Yeo, Sutejo, Tan and Sugrue, unpublished observations]. A gradual increase in the vRNA levels up to 10 hpi (the end point of the experiment) was generally observed following virus infection. At 10 hpi the vRNA level in H5N3 virus-infected A549 cells was approximately 10-fold higher than that measured in H5N2/F118 virus-infected cells, and approximately 100-fold higher than that of the H1N1/WSN virus-infected cells. In H9N2 virus-infected A549 cells the vRNA levels reached a plateau after 1 hr of infection. The H9N2 virus showed the lowest levels of vRNA synthesis in A549 cells, exhibiting a 10,000-fold reduction in vRNA levels compared to that in H5N2/F118 virus-infected cells. In MDCK cells the vRNA levels in both H1N1/WSN and H5N3 virus-infected cells were comparable, being approximately 10-fold and 100-fold higher than that observed in H5N2/F118 and H9N2 virus-infected cells respectively. Although the vRNA levels measured in H5N3 virus-infected CEF cells were approximately 10-fold higher than the other viruses examined, at 10 hpi the vRNA levels in H5N2/F118, H9N2 or H1N1/WSN virus-infected CEF cells were similar. A comparison of the vRNA levels of all AIVs used in this study in each of the three cell types at 10 hpi (Table 1) indicated that the H5N2 viruses behaved similarly, and that the H5N3 and H9N2 virus-infected A549 and MDCK cells exhibited the highest and lowest vRNA levels respectively. In addition, we consistently observed significantly increased vRNA levels for all viruses in CEF cells compared with that recorded in the other cell types.

**Table 1:** Comparison of the M gene vRNA levels at 10 hpi in influenza virus-infected A549, MDCK and CEF.

	vRNA (copy numbers)		
Virus	A549	MDCK	CEF
H1N1	4.43±0.07	5.74±0.07	6.91±0.05
H9N2	1.71±0.04	3.42±0.08	7.34±0.07
H5N2(F118)	5.71±0.04	4.91±0.02	7.47±0.10
H5N2(F189)	5.70±0.08	5.01±0.20	7.28±0.06
H5N3	6.83±0.04	5.93±0.03	8.29±0.06
pH1N1/276	1.92±0.06	2.11±0.26	5.10±0.52
pH1N1/471	1.06±0.09	2.69±0.20	5.27±0.11
pH1N1/478	1.67±0.12	2.53±0.20	4.92±0.33
pH1N1/527	1.32±0.11	2.46±0.18	5.70±0.21

Each cell line was infected with all viruses used in this study at an MOI = 4. The M gene copy numbers are shown per 10^4^ copies of the elongation factor (EF) gene in each host cell line. The vRNA levels are given by a log value. The average value and standard error are shown from triplicate measurements (p<0.05). Representative data from one of two independent experiments is shown.

Influenza virus proteins were examined in ^35^S-methioine-labelled infected cells to confirm the efficiency of translation of the virus mRNA transcripts. Cells were either mock- infected or infected with either the H1N1/WSN, H5N2/F118, H5N3 and H9N2 viruses and at between 9 and 10 hpi the cells were radiolabelled with ^35^[S]-methionine. The radiolabelled cells were examined by SDS-PAGE, and protein bands at approximately 28 kDa and 55 kDa were observed in all lysates prepared from virus-infected cells (Figure 1B); the expected sizes of the matrix (M) protein and nucleoprotein (NP) respectively. As expected, these radiolabelled virus proteins were not observed in the mock-infected ^35^[S]-methionine-labelled cells. Although a small variation in the intensity levels of the various virus protein bands in the different cells was observed (e.g. H9N2 virus-infected A549 and MDCK cells showed a slight reduction in virus protein levels), this analysis suggested that efficient translation of the virus mRNA transcripts had occurred in each cell and virus combination.

In a similar analysis, the replication kinetics in the three cell lines was established for the pH1N1/276, pH1N1/471, pH1N1/478 and pH1N1/527 isolates by performing vRNA quantification at 1 hr intervals up to 10 hpi (Figure 1C). A gradual increase in the vRNA levels up to 10 hpi (the end point of the experiment) was observed in each cell type, however we noted that the four pH1N1 virus isolates replicated less efficiently in A549 and MDCK cells compared to the laboratory isolate H1N1/WSN. An approximate 10,000-fold lower level of vRNA was observed in either pH1N1 virus-infected A549 and MDCK cells compared to that in H1N1/WSN virus-infected cells. We noted higher levels of vRNA in pH1N1 virus-infected CEF cells compared to that in either the A549 or MDCK cells. CEF cells infected with the pH1N1 virus showed only an approximate 50–100 fold lower vRNA levels compared to the H1N1/WSN virus. This suggested that the pH1N1 virus isolates appeared to replicate with a higher efficiency in CEF cells, showing similar replication characteristics to that observed for the H9N2 virus. A comparison of the different pH1N1 viruses with the AIV and H1N1/WSN virus at 10 hpi in all three cell types is shown, suggesting that AIVs examined could replicate in the three cell types (Table 1).

The NP initially accumulates in the nucleus as part of the RNP complex, and it is subsequently exported through the nuclear pores to the sites of virus assembly [18]. The detection of the NP in virus-infected cells is a good indicator of virus gene expression, while its cellular distribution is a good indicator of nuclear export of the ribonucleoprotein (RNP) complex. A549 cells were infected with the H1N1/WSN, H5N2, H5N3 or H9N2, and at specific times after infection the cells were fixed, stained with anti-NP and examined using immune-fluorescence (IF) microscopy (Figure S1). In H1N1/WSN virus-infected A549 cells the NP-staining was restricted to the nucleus at 4 hpi (Figure S1, highlighted by *), which changed to a diffuse (whole cell) staining pattern by 10 hpi (Figure S1, highlighted by white arrow). This change in staining pattern was consistent with nuclear export of the NP by 10 hpi. In contrast, nuclear staining was observed in the H5N2 and H5N3 virus-infected cells by 6 hpi, and by 8 hpi in H9N2 virus-infected cells. However, all AIVs examined exhibited a more prominent nuclear NP-staining. In the case of the H9N2 virus a prominent nuclear staining pattern was still observed by 16 hpi (Figure S1 highlighted by *). In a similar analysis A549 cells were infected with pH1N1/471 or pH1N1/527, and the cells fixed and stained with anti-NP at 5, 10 or 16 hpi (Figure S2). This analysis showed that although anti-NP staining could only be detected by 5 hpi in H1N1/WSN virus-infected A549 cells, both pH1N1 viruses only showed NP staining at 10 hpi. A more detailed analysis showed that both pH1N1 isolates showed extensive NP cytoplasmic staining by 16 hpi.

In H1N1/WSN virus-infected MDCK cells nuclear export was observed at between 4 and 6 hpi (Figure S3), while H5N3 and H9N2 virus-infected cells showed nuclear staining at 6 hpi. In the H5N2 virus-infected cells nuclear staining was observed by 8 hpi. However, all AIV-infected MDCK cells showed a significant degree of nuclear export by 10 hpi. In MDCK cells infected with either pH1N1/471 or pH1N1/527 nuclear staining was apparent by 10 hpi, but by 16 hpi both nuclear and cytoplasmic staining was observed (Figure S4). In CEF cells infected with either of the H1N1/WSN and AIVs NP expression was detected by 6 hpi, and by 10 hpi extensive nuclear export of the NP was apparent in CEF cells infected with either virus (Figure S3). Similarly, CEF cells infected with either pH1N1 virus or H1N1/WSN showed a similar appearance of NP staining by 5 hpi, and by 10 hpi nuclear export of the virus NP was apparent (Figure S4).

Virus-specific differences were observed in RNP complex nuclear export in mammalian cells, while all viruses exhibited similar export characteristics in CEF cells. However all virus and cell combinations exhibited concomitant increase in NP-staining of infected cells as the infection proceeded. Furthermore, examination by IF microscopy confirmed that approximately 95% of cells showed NP staining by 10 hpi in each virus and cell combination, suggesting similar levels of infection in all virus and cell combinations by 10 hpi under our experimental conditions.

Since we noted virus-specific differences in the export of the NP in AIV-infected cells we also examined the infectivity in the tissue culture supernatant (TCS) of H1N1/WSN, H5N2/F118, H9N2 and H5N3 virus-infected cells, which is an indicator of cell-released virus. Near confluent A549, MDCK or CEF cell monolayers were infected with each of the four viruses using an MOI = either 0.1 or 0.01, and at 48 hpi the virus titer in the TCS was determined (Table 2). In A549 cells, cell-free H1N1/WSN virus could only be detected in cells infected using an MOI = 0.1, while extracellular virus particles in the TCS from AIV-infected cells were not detected using either MOI. This clearly suggests that while the H5 virus subtypes in this study could replicate in A549 cells as efficiently as the H1N1/WSN, only the H1N1/WSN virus exhibited significant levels of cell-free virus. In a parallel study A549 monolayers were infected with either the H1N1/WSN or H9N2 virus using an MOI = 0.05 and at 24 hpi the presence of infected cells detected by staining using anti-NP (Figure S5). In H1N1/WSN virus-infected monolayer numerous stained cells were detected using IF microscopy, consistent with the spread of virus to uninfected cells within the A549 cell monolayer. A smaller number of isolated infected cells showing apparent increased intensity of staining were detected in the H9N2 virus-infected monolayer. At later stages in infection (i.e. 36 hpi) these cells detached leaving the intact non-infected monolayer behind [Myaing and Sugrue, unpublished observations]. This suggested that while A549 cells were similarly susceptible to infection with both viruses, subsequent infection of other non-infected cells does not occur following H9N2 virus infection. The H1N1/WSN virus-infected MDCK cells showed the highest virus titer, while the H5N3 or H5N2/F118 viruses showed a 10-fold and 100-fold reduction in virus titer respectively (Table 2). The H9N2 virus-infected MDCK cells exhibited the lowest virus titer, being approximately 500-fold lower that that obtained from H1N1/WSN virus-infected cells. The rate of plaque formation in MDCK cells was also observed over a 7 day period (Figure S6), which showed that the H1N1/WSN virus exhibited the greatest rate of plaque formation, closely followed by the H5N3 virus. Plaque formation by the H5N2/F118 virus was slightly slower than the H5N3 virus, while the H9N2 virus produced visible plaques after 7 days of incubation. In contrast, in CEF virus-infected cells the highest virus titer was recorded in the TCS of H9N2 virus-infected cells, closely followed by the other three viruses. Although these data suggest that the H9N2 virus is able to replicate less efficiently in mammalian cell types, the other AIVs used in this study show equal efficiency of replication compared to the H1N1 viruses. However, the A549 cells infected with either of the AIVs suggest a block in the RNP transport to the sites of assembly at the surface of infected cells.

**Table 2:** Virus titres (pfu/ml) from the tissue culture supernatant of influenza virus-infected MDCK, CEF and A549 cells.

	Cell type					
	MDCK		CEF		A549	
	MOI		MOI		MOI	
Virus	0.1	0.01	0.1	0.01	0.1	0.01
H1N1/WSN	1.7×10^7^	2.2×10^7^	2.2×10^5^	2.8×10^5^	2.0×10^3^	ND
H5N2	2.2×10^5^	8.5×10^5^	4.2×10^5^	4.8×10^5^	ND	ND
H5N3	2.2×10^6^	2.0×10^6^	8.0×10^5^	7.7×10^5^	ND	ND
H9N2	8.5×10^4^	5.7×10^4^	8.5×10^5^	1.1×10^6^	ND	ND

Each cell line was infected with each of the H1N1/WSN, H5N2/F118, H5N3 and H9N2 viruses using an MOI = 0.1 and 0.01 and incubated in DMEM containing 1 µg/ml TPCK trypsin and 0.21% BSA at 37°C. At 48 hpi the virus titres in the tissue culture supernatant were determined by agarose overlay plaque assay on MDCK cells. ND denotes no infectious virus particle detected. Representative data from one of two separate experiments is shown, and the average values are from duplicate measurements (SE<5%).

### Global expression trends in the influenza virus-infected cells

Although all AIVs examined showed comparable levels of infectivity in egg culture, we observed a cell-specific variation in the replication characteristics of these different viruses, suggesting significant differences in the virus and host cell interactions. We therefore used microarray analysis to monitor the effect of virus infection on the host cell transcriptome, since virus-induced changes in the host cell mRNA levels represents a sensitive method to analyse the early effects of virus infection on host cell transcription. The effect of each AIV on the host cell transcriptome in A549, MDCK and CEF cells was examined using the Human HG-U133 Plus 2.0, the Canine Genome 2.0 Array, and the Chicken Genome high density microarray systems respectively. The expression of approximately 40% of the total probe sets on the respective microarray chips could be detected in mock-infected cells, indicating that a relatively large proportion of the total transcriptome in all three cell types was represented in our analysis. Since all four pH1N1 virus isolates showed similar replication properties we used pH1N1/527 as a representative pH1N1 isolate in the microarray analysis. The different cell types have different levels of gene annotation that prevents a direct comparison between the host-cell transcriptome changes, however this approach allows us to compare the host response of the different viruses within the same cell type.

The temporal effect of virus infection in each of the three cell types infected with the H1N1/WSN, pH1N1/527, H5N2/F118 or H9N2 viruses was first examined. The cells were infected with each of the four viruses and at specific time points up to 10 hpi the global host cell transcriptome was analysed. This time interval was sufficient to establish virus infection, but is prior to the extensive cell damage that occurs later in the replication cycle (e.g. following virus-induced cell membrane fusion), cellular changes that are likely to induce gene expression changes not directly related to virus replication. Although the human genome has been extensively annotated and the function of many genes in the GeneChip Human HG-U133 Plus 2.0 system have been defined, many genes in the Canine Genome 2.0 Array and Chicken Genome Array systems are less clearly defined and remain non-annotated. Therefore, a statistical comparison of the global temporal changes in host gene expression was performed using probe set identification, which includes both confirmed and predicted open-reading frames. The transcriptional profile of each cell type varied in a virus-specific manner, and relatively few changes occurred within the first two hours of infection (Figure S7). All virus and cell combinations showed a gradual change in the host cell transcriptome, which correlated with the progress of virus infection (e.g. increased vRNA levels). The greatest rate of change in host gene expression was in general between 4 and 6 hpi, with the highest number of host gene changes being observed at 10 hpi.

In general a larger number of probe sets showing down-regulated expression compared to those showing up-regulated expression was observed (Figure S7). Since the human genome database is relatively well annotated we analysed the proportion of probe sets showing changes in gene expression in A549 virus-infected cells based on gene ontology and functional grouping at 10 hpi (Figures S8 and S9). A larger number of probe sets related to immune response function genes showed a >10 FC increase in expression levels, while a significantly larger number of probe sets related to genes implicated in regulating host gene expression showed >10 FC reduction in expression levels. A significant proportion of the down-regulated gene expression may partly arise due to the cap snatching mechanism employed by influenza for virus gene transcription [19], which would be expected to lead to increased cellular mRNA degradation [20]. However, recent evidence has suggested a role for microRNA (miRNA) expression in controlling the immune response in influenza virus infected [21], and it is not clear to what extent differences in miRNA expression may account for the virus-specific variations in global gene expression that we observed in this study. Furthermore, it is not clear if this down-regulated expression is part of an antiviral response or if down-regulated expression of specific cellular genes and pathways is required for efficient influenza virus replication.

### Cytokine expression and interferon signalling in virus-infected A549 cells

**Figure 2:**
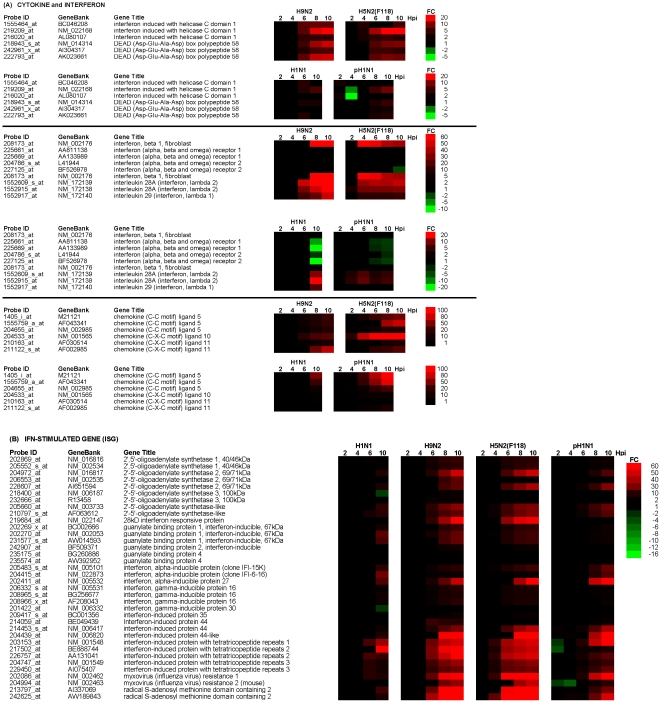
Temporal changes in (A) cytokine and interferon (IFN)-related gene and (B) IFN-stimulated gene (ISG) expression during influenza virus infection. A549 cells were infected with H1N1/WSN, pH1N1/527, H9N2 and H5N2/F118 viruses using an MOI = 4, and at between 2 and 10 hpi the host cell mRNA levels compared with that in mock-infected cells. The data were obtained from 3 independent experiments, and probe sets showing either >2 or <−2 fold change (FC) in expression are indicated (p<0.05). Expression profiles of up-regulated (red), down-regulated (green) and genes showing no change in expression (black) in H1N1/WSN, H9N2 and H5N2/F118 virus-infected A549 cells compared to mock-infected cells are shown. Also shown are the probe identification (probe ID), accession numbers acquired from GeneBank (Gene symbol). DEAD box polypeptide 58 is also known as RIG I protein, while mda5 is also known as IFN-induced helicase C domain-containing protein 1. In addition, the MX1 protein is homologous to the MXA protein in humans.

Although we noted that in general all viruses induced the expression of few cytokines by 10 hpi, cells infected with all AIVs exhibited elevated levels of the cytokines CXCL5 and CXCL11 (Fig. 2). Elevated CXCL10 gene expression levels were observed for all AIVs with the exception of the H5N3 virus. Previous studies have suggested H5N1 virus infection is associated with increased expression of these three cytokines [14], [22]. However, a modified attenuated H5N1 vaccine candidate virus has been produced which down-regulate the expression of these cytokines [22], thus exhibiting properties that are different to both the H5N1 HPAI, and the LPAI viruses examined in this study. This suggests that attenuation of H5N1 HPAI virus by mutation is likely to create virus variants that have distinct properties when compared to the original parent H5N1 virus, and with other circulating LPAI viruses. This highlights the need to examine the biological properties of naturally occurring LPAI viruses.

Interferon (IFN) proteins are important cytokine mediators of the innate immune response, whose expression involves the recognition of specific pathogen-associated molecular patterns (PAMPs) by Pattern recognition receptors (PRRs), such as retinoic acid inducible gene I (RIG-I) and melanoma differentiation-associated gene-5 (mda-5). These include Type I IFN (IFN α/β); Type II IFN (IFNγ); and Type III IFN (IL29, IL28A and IL28B) mediated signalling pathways. In addition, cross-talk between IFN signalling and other signalling pathways that relate to antivirus response have been reported (e.g. JNK signalling), and presumably play a role in the overall antivirus response. IFN induction leads to the phosphorylation and heterodimerisation of the STAT proteins [23], which in turn leads to the transcriptional activation of more than 100 IFN-stimulated genes (ISGs). A significant number of ISGs express proteins that are associated with antivirus activity [24], [25]. Although the three IFN signalling pathways are mediated by different receptor complexes [26], the Type I and Type III IFN proteins activate the same signalling pathways and induce the expression of common ISGs [27], [28], [29], [30].

In H9N2 and H5N2/F118 virus-infected A549 cells a temporal increase in DDX58 (RIG-I) and IFN-induced helicase C domain-containing protein 1 (mda5) expression was observed between 4 and 6 hpi, which correlated with increased IFNβ gene expression from between 4 and 6 hpi up until 10 hpi (Figure 2). This correlated with the increased vRNA synthesis (Figure 1A), suggesting virus replication-dependant changes in IFN expression. The IFNβ gene expression levels in cells infected with either virus were therefore compared at 10 hpi (Table S1), since the temporal analysis suggested that the greatest changes in the IFN expression occurred by 10 hpi. Although in RIG-I and mda-5 expression was also observed in H5N3 and H5N2/F189 virus-infected cells at 10hpi, we noted comparatively smaller increases in IFNβ gene expression than that in H5N2/F118 and H9N2 virus-infected cells. In H1N1/WSN and pH1N1/527 virus-infected A549 cells we observed significantly lower RIG-I and mda-5 expression, and no evidence for increased IFNβ gene expression. Interestingly, in these cases the absence in IFNβ gene expression correlated with reduced expression of the genes encoding the interferon-alpha/beta receptor α (IFNAR1) and β chains (IFNAR2), which together constitutes the IFN type 1 receptor. This data suggests that while the human viruses appear to down-regulate the IFN type I signalling pathway, the AIVs appear to lack this ability. Recent studies have suggested that type I IFN receptor-deficient mice have increased sensitivity to influenza virus infection, suggesting that down-regulated expression of the type I IFN receptor may allow evasion of IFN signalling. This suggests that the ability of these H1N1 viruses to down-regulate the IFN type 1 receptor may form part of virus countermeasure to evade the host cell-antivirus response, which is absent in the LPAI viruses. H5N1 virus is able to down-regulate the expression of IFNAR1 [31] in a similar manner to that observed for the H1N1/WSN and H1N1/2009 viruses in this study, suggesting an additional difference between the H5N1 viruses and the LPAI viruses in this study.

All viruses showed up-regulated IL28A gene expression by 10 hpi, although a virus-specific variation in the expression levels was observed. A role for Type III IFN in suppressing virus infection has been demonstrated [32], suggesting a role for Type III IFN signalling in the antivirus response to influenza virus infection. Similar levels of IL28A and IL29 gene expression were detected in H1N1/WSN, pH1N1/527 and H5N2/F189 virus-infected A549 cells. Interestingly, these observations suggest higher levels of IFN type signalling which is consistent with recent observations for the pH1N1 virus [33]. The highest expression levels were observed in H9N2 and H5N2/F118 virus-infected cells, while the lowest expression levels were observed in H5N3 virus-infected cells. The results of the microarray analysis were supported using qPCR analysis (Figure S10). Although the expression fold-changes were not identical in the microarray and qPCR analysis, the overall trend in gene expression changes was consistent. The general discrepancy in expression fold values that can arise using both methods has been the subject of previous research [34], [35].

**Figure 3:**
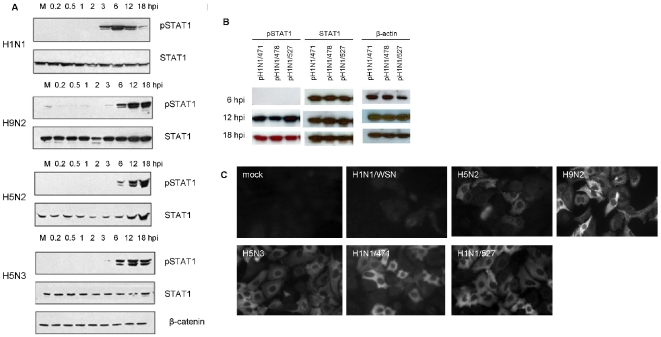
Influenza virus induced changes in STAT1 signalling during virus infection. (A) A549 cells were infected with either the H1N1/WSN, H9N2, H5N2/F118 or H5N3 viruses or (B) the pH1N1/471, pH1N1/478 or pH1N1/527 virus using an MOI = 4. Then cells were harvested at between 0.2 and 18 hpi in SDS-PAGE boiling mix as described in methods. The proteins were transferred on to PVDF membranes by western blotting, and the membranes probed with the appropriate primary and secondary antibodies. The phosphorylated STAT-1 (pSTAT1) and total STAT-1(STAT1) are shown. (A) β-catenin or (B) β-actin provides a loading control. (C) A549 cells were either mock-infected or were infected with the H1N1/WSN, H5N2, H9N2, H5N3, pH1N1/471 or pH1N1/527 viruses using an MOI = 4. At 16 hpi the cells were labelled using anti-MX and goat anti-mouse conjugated to Alexa555. The stained cells were visualised using a Nikon Eclipse 80i Microscope at ×20 magnification using appropriate machine settings.

The microarray analysis suggested increased IFN gene expression during AIV infection, and the activation status of the STAT1 protein was examined. Cells were infected either with the H1N1/WSN, H5N2/118, H9N2 or H5N3 viruses, and the kinetics of STAT1 activation in infected cells determined by detection of phosphorylated STAT1 (pSTAT1) (Figure 3A). STAT1 activation was detected at between 3 and 6 hpi in all virus-infected cells examined. At 18 hpi the signal intensity for pSTAT1 was significantly reduced in H1N1/WSN virus-infected cells, however sustained STAT1 activation was observed in H9N2, H5N3 and the H5N2/F118 virus-infected cells up to 18hpi. A similar analysis of the pH1N1/471, pH1N1/478 and pH1N1/527 virus-infected A549 cells showed activation of STAT1 by 12 hpi (Figure 3B).

The kinetics of STAT1 activation correlated with the expression of several ISGs, although the magnitude of the ISG expression levels varied in a virus-specific manner. The increased expression of several ISGs either with relatively well characterised antiviral activities such as 2′, 5′-oligoadenylate synthase 2 (OAS2), radical S-adenosyl methionine domain containing 2 (RSAD2) and Myxovirus 1 (MX1) was observed. Although recent studies have suggested an antiviral activity associated with IFN-induced protein with tetratricopeptide repeats 1 (IFIT1), and IFN-induced protein with tetratricopeptide repeats 2 (IFIT2) [36], the increased expression of several ISGs with less defined antiviral activities (e.g. IFN-induced protein 44) was also observed (Table S1). In general, the AIVs and pH1N1s showed higher levels of MX and OAS2 gene expressions compared to that in H1N1/WSN virus-infected cells. MX protein expression in virus-infected cells was examined by IF microscopy using anti-MX to detect MXA protein expression in mock-infected or virus-infected A549 cells at 18 hpi. While low level of MXA protein staining was observed in H1N1/WSN infected-A549 cells, significantly higher levels of MXA protein expression was observed in A549 cells infected with the other viruses (Figure 3C). As expected we failed to detect MXA protein expression in mock-infected cells, and this analysis confirmed MXA protein expression in cells infected with either of the AIVs and pH1N1 viruses used in this study. The highest levels of OAS2 gene expression were observed in H9N2, H5N2/F118 and H5N3 virus-infected cells. Interestingly, the H1N1/WSN and H5N3 viruses showed similar and relatively low level of increased RSAD2 gene expression, whereas the other AIVs showed relatively large increases in RSAD2 gene expression. In general we noted that the pH1N1/527 virus-infected cells showed a higher level of ISG expression compared to the H1N1/WSN virus at 10 hpi.

These data suggested that the induction of endogenous IFN expression in AIV-infected A549 cells was sufficient for sustained STAT activation and subsequent induction of ISG expression. Although STAT1 activation was observed in H1N1/WSN, H5N3, H9N2 and H5N2/F118-virus-infected cells, no induction of Type I IFN was observed either by the microarray or qPCR analysis in H1N1/WSN virus-infected cells. Similarly, no induction of Type I IFN was observed in pH1N1/527 virus-infected A549 cells. However, STAT1 activation via Type III IFN signalling has been demonstrated [37], [38]. This suggested that in AIV-infected A549 cells the antivirus response is mediated by a combination of the Type I and Type III IFN signalling pathways, while in H1N1/WSN virus-infected cells the Type III IFN signalling pathway may be more important. Although the importance of Type 1 IFN signalling in determining pathogenicity has been demonstrated [39], a commitment increase in both Type I and Type III IFN signalling enhances the antivirus response following influenza virus infection in mice . Several of the ISGs identified in our study can be induced via Type I and Type III signalling pathways [30], which may account for the generally high level of ISG expression in the AIV-infected cells.

Temporal analysis of the microarray data obtained using H5N2/F118 virus-infected cells suggested that increased IL28A expression occurred concomitantly with IFNβ gene expression, while increased expression of these genes in H9N2 virus-infected cells occurred slightly later. These temporal differences in IFN expression correlated with the different rates of vRNA synthesis in A549 cells. The presence of vRNA (e.g. the 5′ terminal phosphate) is proposed to play a role in inducing IFN signalling and is largely responsible for initiating the antivirus response [40], [41]. Although poor replication of the H9N2 virus in A549 cells was observed, similar levels of up-regulated ISG expression were observed when compared with other viruses, suggesting that the vRNA levels in the AIV-infected cells may be above a certain threshold level to induce IFN signalling. Moreover, the mechanism of action for many of the putative antivirus ISGs identified in this study are poorly defined e.g. IFIT1, and it is not clear if the replication characteristics of the AIVs that we observed in these cells is directly related to the expression of one or more these ISGs.

### Interferon signalling in virus-infected MDCK and CEF cells

**Figure 4:**
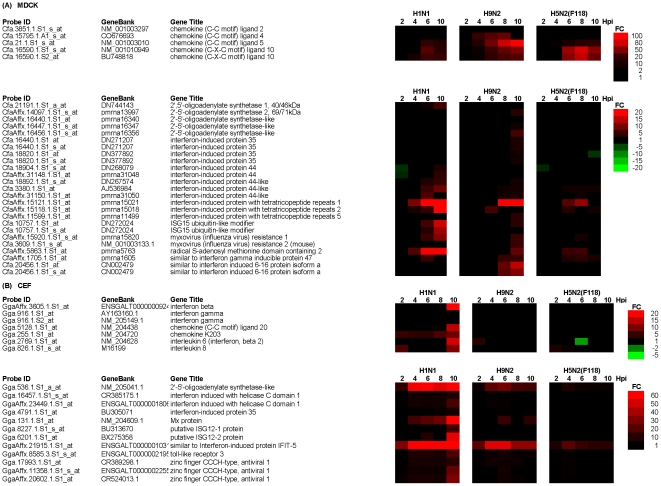
Temporal changes in the expression levels of cytokine and interferon (IFN) and IFN-stimulated genes (ISG) in influenza virus-infected (A) MDCK and (B) CEF cells. Cells were infected using an MOI = 4, and at between 2 and 10 hpi the host cell mRNA levels compared with that in mock-infected cells. The data were obtained from 3 independent experiments, and probe sets showing either >2 or <−2 fold change (FC) in expression are indicated (p<0.05). In this representation up-regulated (red) or down-regulated (green) refer to the fold changes (FC) in gene expression compared to mock-infected cells in H1N1/WSN, H9N2 or H5N2/F118 virus-infected cells. Also shown are the probe identification (probe ID), accession numbers acquired from GeneBank (Gene symbol) and gene name (Gene title).

The temporal change in host gene expression was examined in H1N1/WSN, H9N2 and H5N2/F118 virus-infected MDCK and CEF cells up until 10 hpi (Figure 4). In virus-infected MDCK cells we failed to detect significant levels of increased either RIG-I or mda-5 expression and this correlated with the absence of IFNβ expression. Furthermore, either no or relatively small increases in MX and RSAD2 gene expression were observed. However, relatively high expression levels of other putative ISGs were observed in H1N1/WSN virus-infected cells, which included IFN-induced protein 44-like, IFIT1, IFIT2, and IFN-stimulated gene-15 (ISG15). A comparison of the expression levels at 10 hpi suggested that in general the expression of these genes were highest in H1N1/WSN virus-infected cells compared to that in cells infected with either of the AIVs (Table S12). In addition, we noted that with the exception of the H5N3 virus, all viruses examined induced CCL5 and CCL10 gene expression.

In H1N1/WSN virus-infected CEF cells we observed increased expression of several cytokines at between 8 and 10 hpi. In addition, a temporal increase in mda-5 expression was observed at 4 hpi, and increased IFNβ gene expression was observed at between 8 and 10 hpi. This correlated with increased expression of several ISGs, including 2′, 5′-oligoadenylate synthase-like (OASL) and MX gene expression. Although no IFNβ gene expression was observed in AIV-infected cells, low levels of ISG expression were observed. However, the anti-virus response was significantly higher in the H1N1/WSN virus-infected CEF cells compared to all the AIVs examined (Table S3). We noted that although the AIVs grew equally well in CEF cells we failed to observe a robust IFN induction. RIG-I is a cytoplasmic RNA sensor that detects the presence of vRNA, leading to induced expression of IFN-β and downstream ISGs [42], [43]. Recent evidence has suggested that a factor in the susceptibility of chickens to AIV infection may be the absence of RIG-I expression [44]. Since CEF cells are derived from chickens this may explain the absence of induced IFNβ gene expression in the AIV-infected CEF cells. However, we observed increased expression of IFNβ in H1N1/WSN-infected CEF cells, suggesting that these viruses may induce IFN signalling via an alternative pathway. The qPCR analysis confirmed increased MX and OASL gene expression in the H1N1/WSN virus-infected CEF cells (Figure S10). Increased gene expression of the putative ISG IFIT-5 was detected in all viruses, which is consistent with recent observations in AIV-infected macrophages which indicated increased IFIT-5 gene expression even in the absence of increased IFN gene expression [45]. In general expression trends observed in CEF cells was the converse of that observed in the A549 cells, suggesting a more potent antivirus response in CEF cells infected with the H1N1/WSN virus compared to the AIVs.

### Induction of cell death signaling in virus-infected A549 cells

**Figure 5:**
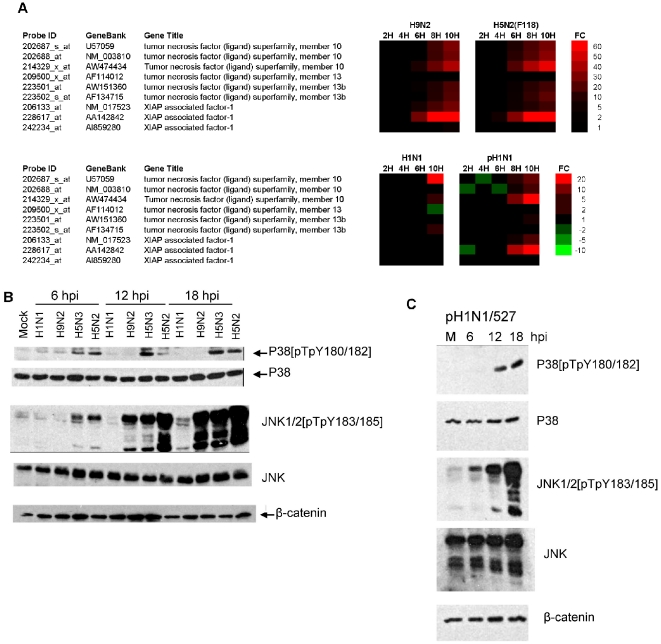
Temporal changes in the expression of selected cell death signalling related genes during influenza virus infection. (A). A549 cells were infected with H1N1/WSN, pH1N1/527, H9N2 and H5N2/F118 virus using an MOI = 4, and at between 2 and 10 hpi the host cell mRNA levels compared with that in mock-infected cells. The data were obtained from 3 independent experiments, and probe sets showing either >2 or <−2 fold change (FC) in expression are indicated (p<0.05). Expression profiles of up-regulated (red), down-regulated (green) and genes showing no change in expression (black) compared to mock-infected cells in H1N1/WSN, H9N2 and H5N2/F118 virus-infected A549 cells are shown. Also shown are the probe identification (probe ID), accession number (GeneBank) and gene name (Gene title). A549 cells were infected with (B) H1N1/WSN, H9N2, H5N2/F118 or H5N3 viruses or (C) pH1N1/527 using an MOI = 4 and the cells were harvested at the various time-points post-infection using SDS-PAGE boiling mix as described in methods. The proteins were transferred on to PVDF membranes by western blotting, and the membranes probed with the appropriate primary and secondary antibodies. Total p38 (P38), phosphorylated P38 (p38[pTpY180/182]), total JNK (JNK) and phosphorylated JNK (JNK1/2[pTpY183/185]) is shown. Immunoblotting using anti β-catenin provides a loading control.

In general few changes in the expression of genes that are associated with cell death were observed in virus-infected A549 cells. However, a temporal increase in the expression of a small number of cell death related genes, including XIAP associated factor 1 (XAF1), were observed in H9N2, pH1N1/527 and H5N2/F118 virus-infected A549 cells (Figure 5A). XAF1 is able to regulate apoptosis by abrogating the anti-apoptotic activities of the XIAP [46], [47]. Although all the AIVs exhibited relatively strong XAF1 gene expression by 10 hpi (Table S1), increased XAF1 gene expression in H1N1/WSN virus-infected cells was not observed.

Activation of c-Jun N-terminal kinase (JNK) and P38 mitogen-activated protein kinase (p38 MAPK) can be mediated via Type I and Type III IFN signalling pathways [30], [48], [49], [50], and activation of RNase L by OAS gene products have been implicated in p38 and JNK activation [51], [52]. XAF1 expression is enhanced by activated JNK [53], and JNK and p38 MAPK activation has been shown to play a role in the host response to influenza virus infection [54], [55]. The activation status of these pathways in virus-infected A549 cells were examined by immunoblotting using appropriate antibodies to detect the presence of phosphorylated JNK and p38 MAPK (Figure 5B). Similar levels of phophosphorylated p38 MAPK was observed at between 6 and 18 hpi in H5N2/F118 and H5N3 virus-infected cells, but activated p38 MAPK was not detected either in mock-infected cells, or in cells infected with either the H1N1/WSN or H9N2 viruses. Although activated JNK was not detected in H1N1/WSN virus-infected cells, sustained JNK activation up until 18 hpi was observed in H5N2/F118 and H5N3 virus-infected cells from 6 hpi, and from 12 hpi in H9N2 virus-infected cells. Similarly, we observed sustained JNK and p38 MAPK activation in pH1N1/527 virus-infected A549 cells at 6 and 12 hpi respectively (Figure 5C). The absence of JNK activation in H1N1/WSN virus-infected cells is consistent with the absence of increased XAF1 gene expression. A role for JNK activation in regulating apoptosis has been proposed [56], and the correlation between JNK activation and enhanced expression of XAF1 in AIV-infected cells suggests a role for JNK activation in the induction of cell death pathways during AIV infection. In AIV-infected MDCK and CEF cells most genes associated with cell death either showed no change or a reduction in gene expression levels (Tables S3 and S4).

Type I IFN signalling pathway is an important first line of defence against influenza virus infection, and the Type III IFN pathway can also contribute to the overall strength of the innate immune response [57]. In H1N1 virus-infected cells we noted the absence of any significant increased Type I IFN expression and only moderate increases in Type III IFN expression, consistent with recent observations [14]. HPAI virus infection attenuates the IFN response in infected epithelial cells [58], and the NS1 protein has been shown play an important an important to role in overcoming the IFN-mediated antivirus response via an interaction with RIG-I. The NS1 protein prevents Type I IFN induction of an antiviral state during HPAI H5N1 virus infection, by impairing the phosphorylation and activation of the STAT proteins [31]. The LPAI viruses in our study exhibited some distinct characteristics to that described for the H5N1 virus. The LPAI viruses examined in this study appear to be potent inducers of both Type I and Type III signalling pathways, leading to the sustained phosphorylation of the STAT proteins and ISG expression. In addition, we noted that while the H1N1 viruses examined in this study showed down-regulated expression of the IFN type I receptor, the expression levels of the IFNAR1 and IFNAR2 genes remained unchanged in LPAI virus-infected cells. The down-regulated expression of at least one of the components of the IFNα/β receptor during H5N1 virus infection has been described, and the NS1 protein was implicated in down regulating the expression of IFNAR1 [31]. Therefore the NS1 protein may be a primary determinant in overcoming the IFN response during AIV virus infection, and differences in the activity of the NS1 protein in H5N1 virus and the LPAI viruses may account in for differences in IFN induction. This biological activity may be related to the structural characteristics of the NS1 protein that has been reported to form the H5N1 virus, which forms an unusual tubular structures compared with the NS1 protein of other influenza viruses that have been examined [59]. The replication characteristics of the AIVs in mammalian cells are consistent with their replication characteristics in BalbC mice. All the LPAI viruses could infect these animals but they exhibited low levels of virus replication in the lung, and the mice remained asymptomatic. In contrast H1N1 viruses used in this study were capable of inducing lethal infection in mice (Yeo, Tan and Sugrue unpublished observations).

Although all AIVs exhibited differences in their replication characteristics in mammalian cell types, our data does suggest that the H5N2 and H5N3 viruses could efficiently replicate in A549 and MDCK cells. This suggests that these viruses can adapt to replicate in non-avian cell types. All AIVs examined in this study were potent inducers of IFN signalling and we can speculate that a robust IFN response in LPAI virus-infected cells may be one factor that attenuates these viruses in mammalian hosts. The expression of one or more ISG may be related to the nuclear accumulation of the RNPs in the AIV-infected A549 cells, suggesting a block in the RNP export. Although it is currently not clear to what extent the induction of ISG expression is involved in creating this phenotype, the MXA protein can interact with the nucleocapsids of several viruses, inhibiting the translocation of viral components between the nucleus and cytoplasm [60], [61]. In this context an interaction between the MXA and NP has been demonstrated [62]. Although these changes in ISG expression correlate with the attenuation of the virus replication in A549 cells other aspects of the IFN response may also be involved. In addition to ISG expression, the interplay between IFN signalling and cell death signalling has been reported. We noted a correlation between IFN induction and the gene expression changes that are associated with increased apoptosis in the AIV-infected cells. The induction of apoptosis in epithelial cell infected with the H5N1 virus has been reported [63], [64], suggesting that this may be a common property among AIVs that infect mammalian cells.

More efficient replication in cells with an avian background was a common trend among all viruses examined. Interestingly, although the AIVs replicated efficiently in the CEF cells we obtained no evidence for significant increases in IFN production, while increased IFNβ expression was observed in H1N1/WSN virus-infected cells. Since replication efficiency may partly be dictated by these cellular interactions, this suggests differences in cellular interactions may occur in sequence-specific manner. The association of cellular proteins with mature influenza virus particles has been demonstrated [65], and this suggests that a complex network of interactions exist between several virus and cellular proteins, and by extrapolation between virus proteins and cell signalling networks [66], [67], [68]. The observed different replication efficiencies and differences in host response to infection between the different viruses used in this study presumably reflect sequence variations in these viruses that may influence pathogen-host interactions. The observed differences in the replication characteristics and antivirus signalling responses among the LPAI and H1N1 viruses in this study highlight the importance of examining the host response to influenza viruses that have not been extensively adapted to mammalian tissue culture.

## Materials and Methods

### Cells, antibodies and virus culture

Human alveolar basal epithelial (A549, ECACC 86012804, from European Collection of Cell Cultures) and Madin-Darby canine kidney (MDCK, ECACC 84121903, from European Collection of Cell Cultures) cells were maintained in Dulbecco's Modified Eagle's medium (DMEM) (Invitrogen) with 10% FBS and 1% penicillin/streptomycin (pen/strep) (Invitrogen). Chick embryo fibroblasts (CEF) were prepared from 8 to 10 day-old chick embryos and maintained in DMEM containing 10% FBS and pen/strep. The LPAI isolates A/Duck/Malaysia/F118/2004 (H5N2/F118), A/Duck/Malaysia/F189/2004 (H5N2/F189), A/Duck/Malaysia/F59/2004, A/Duck/Singapore-Q/F119/1997 (H5N3) and A/Duck/Malaysia/02/2001 (H9N2) were obtained from the Agri-Food and Veterinary Authority of Singapore and have been described previously [15]. The viruses A/Singapore/2009 (pH1N1) viruses were isolated from patients during the influenza virus pandemic in 2009. The viruses isolates A/Singapore/276/2009(H1N1) (pH1N1/276), A/Singapore/471/2009(H1N1) (pH1N1/471), A/Singapore/478/2009(H1N1) (pH1N1/478), and A/Singapore/527/2009(H1N1) (pH1N1/527) were obtained by culturing nasopharyngeal washings in MDCK cells, after which the tissue culture supernatants were cultured once in embryonated eggs. The laboratory-adapted A/WSN/1933 (H1N1/WSN) (VR-1520) was purchased from American Type Culture Collection (ATCC). All virus stocks were prepared in 9 to 11-day-old embryonated chicken eggs, and the infectivity assessed using standard overlay plaque assay or by determining the TCID_50_ in MDCK cells. Virus infections in A549, MDCK and CEFs were carried out in Dulbecco's Modified Eagle's medium (DMEM) (Invitrogen) with 2% FBS and pen/strep at 37°C in 5% CO_2_. Virus was allowed to absorb to the cell monolayer for 1 hr at 37°C, after which it was removed and replaced with prewarmed DMEM (with 2%FCS with pen/strep). The NP (Chemicon, USA), STAT1, STAT1 pY701 (BD Transduction Technology), JNK 1 & 2 [pT183/pY185] antibody was purchased from Biosource, total JNK, total p38α and p38α [pT180/pY182] antibodies were purchased from Cell Signaling, and β-catenin antibody was purchased from Santa Cruz. The MX antibody was obtained from Georg Kochs (University of Freiburg).

### Plaque assay

Near confluent MDCK cell monolayers in 35 mm dishes were incubated with serial dilutions of virus inoculum (prepared using PBS) at 37°C for 1 hr. The inoculums were removed and replaced with 1% LMP agarose (Sigma) containing DMEM with 1 µg/ml TPCK trypsin and 0.21% BSA at 37°C. Once the agarose had solidified the dishes were incubated in a humidified chamber at 37°C with 5% CO2.

### SDS PAGE and Western blotting

Cell lysates were prepared in 1× boiling mix (1% SDS, 15% glycerol, 1% β-mercaptoethanol, 60 mM sodium phosphate, pH 6·8) and heated at 100°C for 2 min. The cells were radiolabelled with ^35^[S]-methionine at 100 µCi/ml, and the protein sample separated by SDS–PAGE. After SDS-PAGE the polyacrylamide gels were fixed in 10% acetic acid and vacuum-dried. Radiolabelled protein bands were detected by exposing the dried gel to X-ray film (Fuji Photo Film Co Ltd, Japan) at −70°C. In western blotting, the proteins were transferred on to PVDF membranes using the mini blotting apparatus (BioRad, USA), after which the membranes were washed with PBSA and blocked for 18 hr at 4°C in PBSA containing 1% BSA and 0.05% Tween 20. The membrane was incubated with the specific primary antibody, followed by the appropriate anti-mouse or anti-rabbit IgG (whole molecule) peroxidase conjugate (Sigma, USA). The protein bands were visualized using the ECL protein detection system (Amersham, USA). In all cases the apparent molecular masses were estimated using Kaleidoscope protein standards (Bio Rad, USA).

### Immunofluorescence microscopy

Briefly, cells on 13 mm glass cover slips were fixed with 3% paraformaldehyde in PBS and permeabilised using 0.1% saponin. The cells were labelled with anti-NP (Chemicon, USA) and anti-mouse IgG conjugated to FITC (Chemicon, USA). The stained cells were mounted on slides using Dakocytomation (Dako, USA) and visualized either using a Nikon eclipse 80i fluorescence microscope, or a Zeiss Axioplan 2 LSM510 confocal microscope using appropriate machine settings. Confocal microscopy images were processed using LSM510 software.

### Quantitative Real-time quantitative PCR (qPCR)

Total RNA was extracted from cells at 4°C using the RNeasy kit (Qiagen, USA) and reverse-transcribed using Superscript II (Invitrogen, USA) according to manufacturer's instructions. Primers for cell-specific genes were designed using the Probefinder software (http://qpcr.probefinder.com/organism.jsp) from the Universal Probe Library (UPL) Design Center (Roche). Quantitative Real-time PCR (qPCR) was carried out with the iCycler System (BioRad) following the protocol previously described [17]. The sequences of the elongation factors (EF) EEF1A1 (*H. sapiens*) (Genebank Accession Number NM_001402), EEF1A1 (*G. gallus*) (Genebank Accession Number NM_204157) and EEF1A2 (*C. lupus familiaris*) (Genebank Accession Number NM_531877) were used as the reference genes since their expression were validated as being “not significantly changed” throughout all observed time points in the microarray analyses (P-value<0.05). Both absolute and relative quantification analysis were done using comparative Ct (ΔΔCt method) [69]. Standard curves for M and EF were generated and the number of copies of M for each virus was calculated relative to 10^4^ copies of corresponding cell line's EF gene. Relative fold-change of the host virus gene expression were calculated with respect to the mock-infected cells and normalized with the corresponding cell line's EF gene. Primers and probes used for the virus M and host EF gene are shown in Table S4. The statistical analysis was performed on single and paired samples as appropriate by applying the student t-test using a significance cut-off of p<0.05.

### Microarray experiment and data analysis

MDCK, A549 and CEF monolayers were either mock-infected or virus-infected at an MOI = 4. At specific time intervals the cells were harvested in RNAlater (Ambion, USA) and PBS buffer (1∶1), aliquoted, pelleted and stored at −80°C before being processed for microarray analysis. Each analysis was performed from three independent experiments. Total RNA was extracted from approximately 1×10^7^ cells using the RNeasy MiniKit (Qiagen, USA) and quantified using the Nanodrop ND-1000 Spectrophotometer (Thermo Fischer Scientific, USA). Double-stranded cDNA was synthesized from 3 µg of total RNA with the GeneChip One-Cycle cDNA synthesis kit (Affymetrix, USA), followed by synthesis of biotin-labelled cRNA using the GeneChip IVT labelling kit (Affymetrix, USA), according to standard Affymetrix protocols. After cRNA fragmentation, 15 µg of biotin-labelled cRNA was hybridized to the GeneChip Canine Genome 2.0 Array (Affymetrix, USA), the GeneChip Chicken Genome Array (Affymetrix, USA) and Genechip Human Genome HG U133 Plus 2.0 Array (Affymetrix, USA) as appropriate to the host cell line being analyzed. The arrays were washed and stained using the Hybridization, Wash and Stain Kit (Affymetrix, USA) and the GeneChip Fluidic Station 450 (Affymetrix, USA) according to the standard Affymetrix protocols. Finally, the arrays were scanned with the GeneChip scanner 3000 (Affymetrix, USA). Affymetrix CHP files were generated from GeneChip Operating Software (GCOS) version 5.0 and subsequently imported into GeneSpring GX (version. 11) for analysis for each host cell line. Normalization was performed using the RMA method, followed by normalization to specific probe sets on the array as recommended by Affymetrix, which also include the hybridization control *bioB*, *bioC*, *bioD* and *cre*. The signal from mock-infected cells was used as a reference point against which to measure fold-change. Only probe sets that were flagged “present” in at least half of the data sets were considered for analysis, and probe sets were considered as statistically and significantly changed if the P-value of the student t-test and the Benjamini-Hochberg False Discovery method was less than 0.05. Significantly up-regulated and down-regulated probes were defined by a 2-Fold Change (FC) with respect to the mock-infected, and P<0.05. Probe sets were grouped based on their biological function and cellular component as annotated by Gene Ontology (GO) SLIMS. All microarray data was deposited as MIAME-compliant data submissions (GSE31469-72; GSE31474-6; GSE31499; GSE31501; GSE31505-6; GSE31508-12; GSE31514; GSE31516; GSE31518) in the Gene Expression Omnibus.

## Supporting Information

Figure S1 **Analysis of the RNP nuclear export in AIV-infected A549 cells.** A549 cells were infected with either the H1N1/WSN, H9N2, H5N2/F118 or H5N3 viruses using an MOI = 4. At specific times post infection the cells were fixed and labelled using anti-NP and goat anti-mouse conjugated to Alexa555. The stained cells were visualised using a Nikon Eclipse 80i Microscope at ×20 magnification with appropriate machine settings. The NP-stained nuclei (*) and cells (white arrow) are indicated.
(TIF)

Figure S2 **Analysis of the RNP nuclear export in pH1N1 virus-infected A549 cells.** Cells were infected with either the H1N1/WSN, pH1N1/471 or pH1N1/527 viruses using an MOI = 4. At specific times post infection the cells were fixed and labelled using anti-NP and goat anti-mouse conjugated to Alexa555. The stained cells (highlighted by white arrow) were visualised using a Nikon Eclipse 80i Microscope at ×20 magnification with appropriate machine settings.
(TIF)

Figure S3 **Analysis of the RNP nuclear export in AIV-infected MDCK and CEF cells.** The cells were infected with either the H1N1/WSN, H9N2, H5N2/F118 or H5N3 viruses using an MOI = 4, and at specific times post infection the cells were fixed and labelled using anti-NP and goat anti-mouse conjugated to Alexa555. The stained cells were visualised using a Nikon Eclipse 80i Microscope at ×20 magnification with appropriate machine settings. The NP-stained nuclei (*) and cells (white arrow) are indicated.
(TIF)

Figure S4 **Analysis of the RNP nuclear export in pH1N1 virus-infected MDCK or CEF cells.** Cells were infected with either the H1N1/WSN, pH1N1/471 or pH1N1/527 viruses using an MOI = 4. At specific times post infection the cells were fixed and labelled using anti-NP and goat anti-mouse conjugated to Alexa555. The stained cells were visualised using a Nikon Eclipse 80i Microscope at ×20 mag with appropriate machine settings. The stained cells are indicated (white arrow).
(TIF)

Figure S5 **Cell-to-cell transmission in H1N1/WSN and H9N2 virus-infected A549 cell monolayers.** Near confluent A549 cell monolayers were infected with either the H1N1/WSN or H9N2 viruses using an MOI = 0.01 in DMEM containing 1 µg/ml TPCK trypsin and 0.21% BSA at 37°C. At 24 hpi the cells were fixed and labelled using anti-NP and anti-mouse IgG conjugated to FITC. The labelled cells were viewed using a fluorescence microscope and the nuclei are highlighted (white arrow). Insets show the staining pattern at higher magnification.
(TIF)

Figure S6 **Plaque formation in MDCK cells infected with influenza virus.** Near confluent MDCK cells were infected either with the H1N1/WSN, H5N2/F118, H5N3 or H9N2, and virus plaque formation monitored using an agarose overlay plaque assay over a 7 day period. The start of plaque formation (indicated by the black arrow) and the centre of the final plaque (*) imaged at 7 days post-infection are highlighted.
(TIF)

Figure S7 **Temporal changes in the host cell transcriptome during influenza virus infection.** A549, CEF and MDCK cell monolayers were either mock-infected or infected with H1N1/WSN, pH1N1/527, H5N2/F118 and H9N2 using an MOI = 4. The global host gene expression profiles in virus-infected cells were compared to mock-infected cells by microarray analysis. The fold change in gene expression in virus-infected cells is shown at each time point examined. The data were obtained from 3 independent experiments, and probe sets showing either >2 or <−2 fold change (FC) in expression are indicated (p<0.05). Expression profiles of up-regulated (red), down-regulated (green) and genes showing no change in expression (black) are shown. The insets are expanded areas showing the up-regulated and down-regulated probe sets between 2 and 10 hpi, and the colour range indicating the fold change range is also shown.
(TIF)

Figure S8 **Overview of selected gene families, showing significantly up-regulated expression in A549 cells infected with influenza viruses.** A549 cells were infected with either the H1N1/WSN, H9N2, H5N2/F118, H5N2/189, H5N3 or pH1N1/527 viruses at an MOI = 4 and analysed at 10 hpi. The proportion of probe sets in the different gene families, including non-annotated and unclassified gene groups, showing greater than 2-fold change in gene expression and those showing greater than 10-fold change in gene expression are presented. These are the results of 3 independent experiments, where probe sets showing either >2 or <−2 fold change (FC) in expression are indicated (p<0.05).
(TIF)

Figure S9 **Overview of selected gene families, showing significantly down-regulated expression in A549 cells infected with influenza viruses.** A549 cells were infected with either the H1N1/WSN, H9N2, H5N2/F118, H5N2/189, H5N3 or pH1N1/527 viruses at MOI = 4 and at 10 hpi, were analysed. The proportion of probe sets in the different gene families, including non-annotated and unclassified gene groups, showing less than −2-fold change in gene expression and those showing less than −10-fold change in gene expression are presented. These are the results of 3 independent experiments, where probe sets showing either >2 or <−2 fold change (FC) in expression are indicated (p<0.05).
(TIF)

Figure S10 **Relative expression of the selected interferon (IFN) and IFN-stimulated genes (ISGs) as measured by qPCR.** A549, MDCK and CEF cells were infected with either H1N1/WSN, H9N2, H5N2/F118 or H5N3 viruses at an MOI = 4, and analysed at 10 hpi. The average values and standard error were obtained from three independent experiments (p<0.05). IFN-β1: interferon β; MX1: myxovirus resistance 1; OAS1: 2′, 5′-oligoadenylate synthase 1; OAS2: 2′, 5′-oligoadenylate synthase 2; OASL: 2′, 5′-oligoadenylate synthase-like; RSAD2: radical S-adenosyl methionine domain containing 2; IL28: interleukin 28 (interferon λ2); BIRC4BP: XIAP associated factor 1 (XAF1).
(TIF)

Table S1 **Interferon, selected ISG and host genes involved in cell death pathways in influenza virus-infected A549 cells.** A FC value of 1 indicates not significantly changed. These are the results of 3 independent experiments, (p<0.05).
(TIF)

Table S2 **Comparison of gene expression values of selected ISGs in influenza virus-infected MDCK cells.** A FC value of 1 indicates not significantly changed. These are the results of 3 independent experiments, (p<0.05).
(TIF)

Table S3 **Comparison of gene expression values of IFN and selected ISGs in influenza virus-infected CEF cells.** A FC value of 1 indicates not significantly changed. These are the results of 3 independent experiments, (p<0.05).
(TIF)

Table S4 **Primer and probes sequences designed for real-time qPCR.** Primer sequences and UPL probes (Roche) used for real-time qPCR validation of influenza M gene segment and selected canine, chicken and human host genes. IFN-β1: interferon β; MX1: myxovirus resistance 1; OAS1: 2′, 5′-oligoadenylate synthase 1; OAS2: 2′, 5′-oligoadenylate synthase 2; OASL: 2′, 5′-oligoadenylate synthase-like; RSAD2: radical S-adenosyl methionine domain containing 2; IL28: interleukin 28 (interferon λ2); BIRC4BP: XIAP associated factor 1 (XAF1); EF: elongation factor.
(TIF)
