# Supplementary figures and images for: Activation of Type I and III Interferon Signalling Pathways Occurs in Lung Epithelial Cells Infected with Low Pathogenic Avian Influenza Viruses
Source: PLoS One. 2012 Mar 21;7(3):e33732. doi: 10.1371/journal.pone.0033732 (PMC3312346; doi:10.1371/journal.pone.0033732)

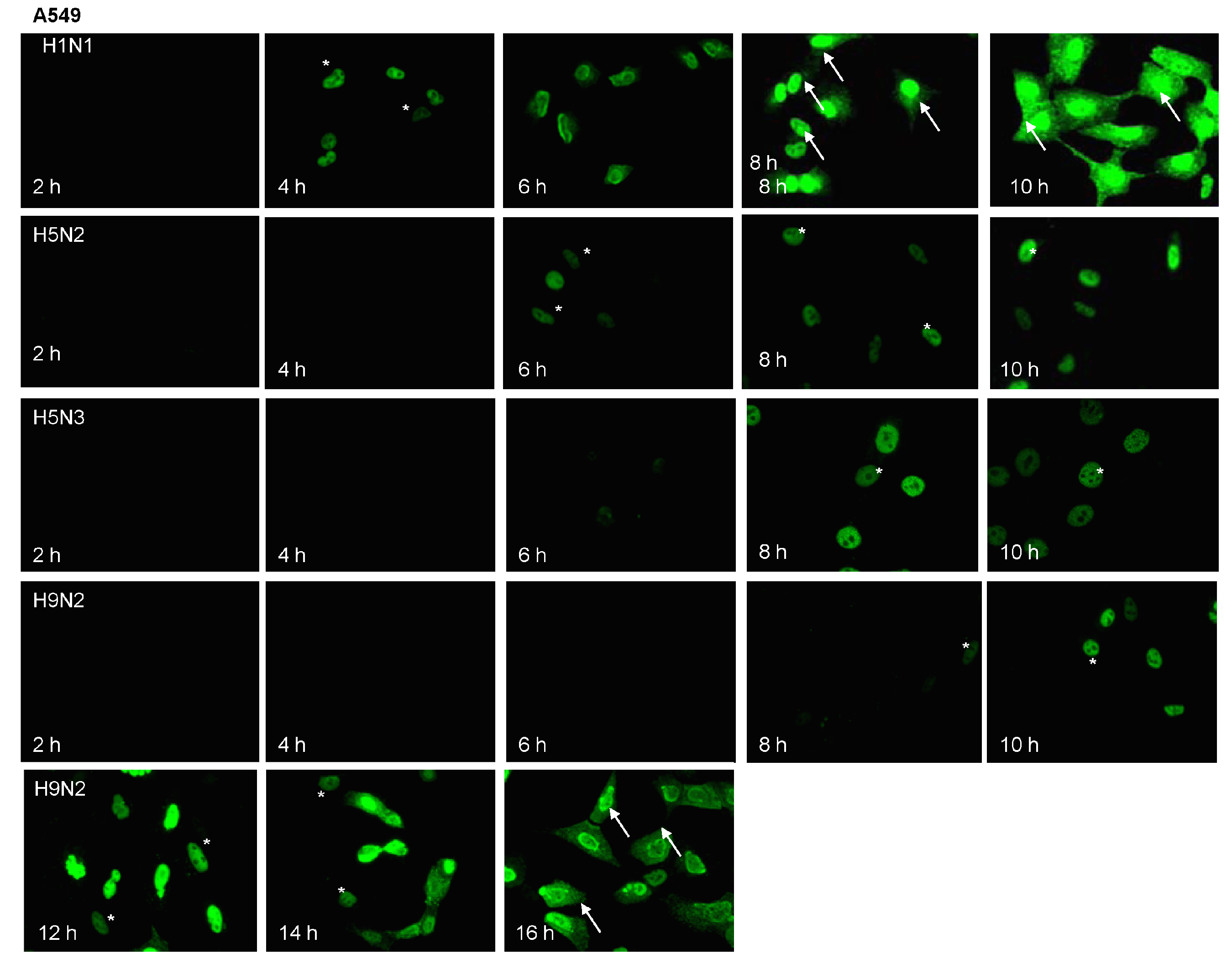

Supplement: Figure S1 — Analysis of the RNP nuclear export in AIV-infected A549 cells. A549 cells were infected with either the H1N1/WSN, H9N2, H5N2/F118 or H5N3 viruses using an MOI = 4. At specific times post infection the cells were fixed and labelled using anti-NP and goat anti-mouse conjugated to Alexa555. The stained cells were visualised using a Nikon Eclipse 80i Microscope at ×20 magnification with appropriate machine settings. The NP-stained nuclei (*) and cells (white arrow) are indicated. (TIF) [file pone.0033732.s001.tif]

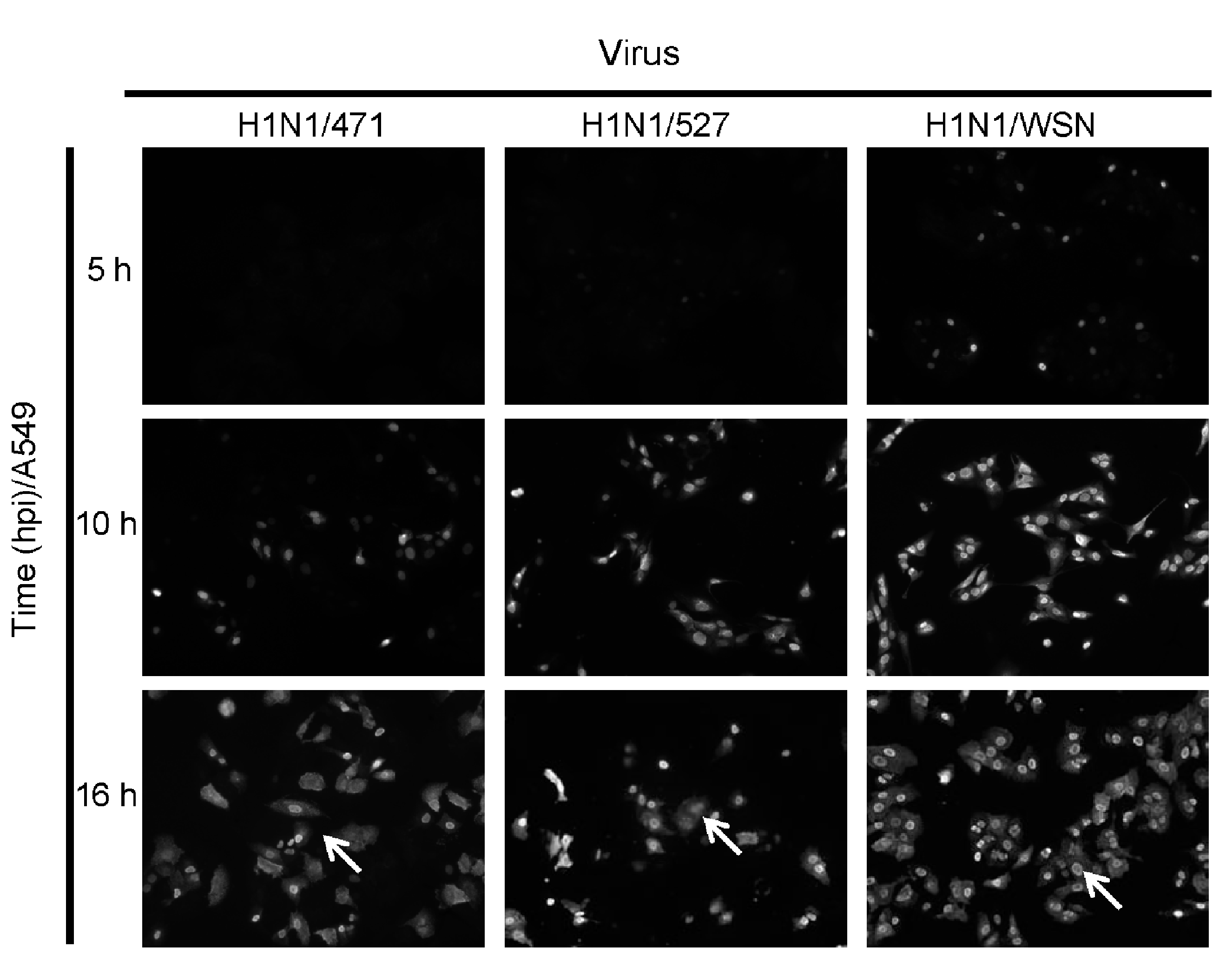

Supplement: Figure S2 — Analysis of the RNP nuclear export in pH1N1 virus-infected A549 cells. Cells were infected with either the H1N1/WSN, pH1N1/471 or pH1N1/527 viruses using an MOI = 4. At specific times post infection the cells were fixed and labelled using anti-NP and goat anti-mouse conjugated to Alexa555. The stained cells (highlighted by white arrow) were visualised using a Nikon Eclipse 80i Microscope at ×20 magnification with appropriate machine settings. (TIF) [file pone.0033732.s002.tif]

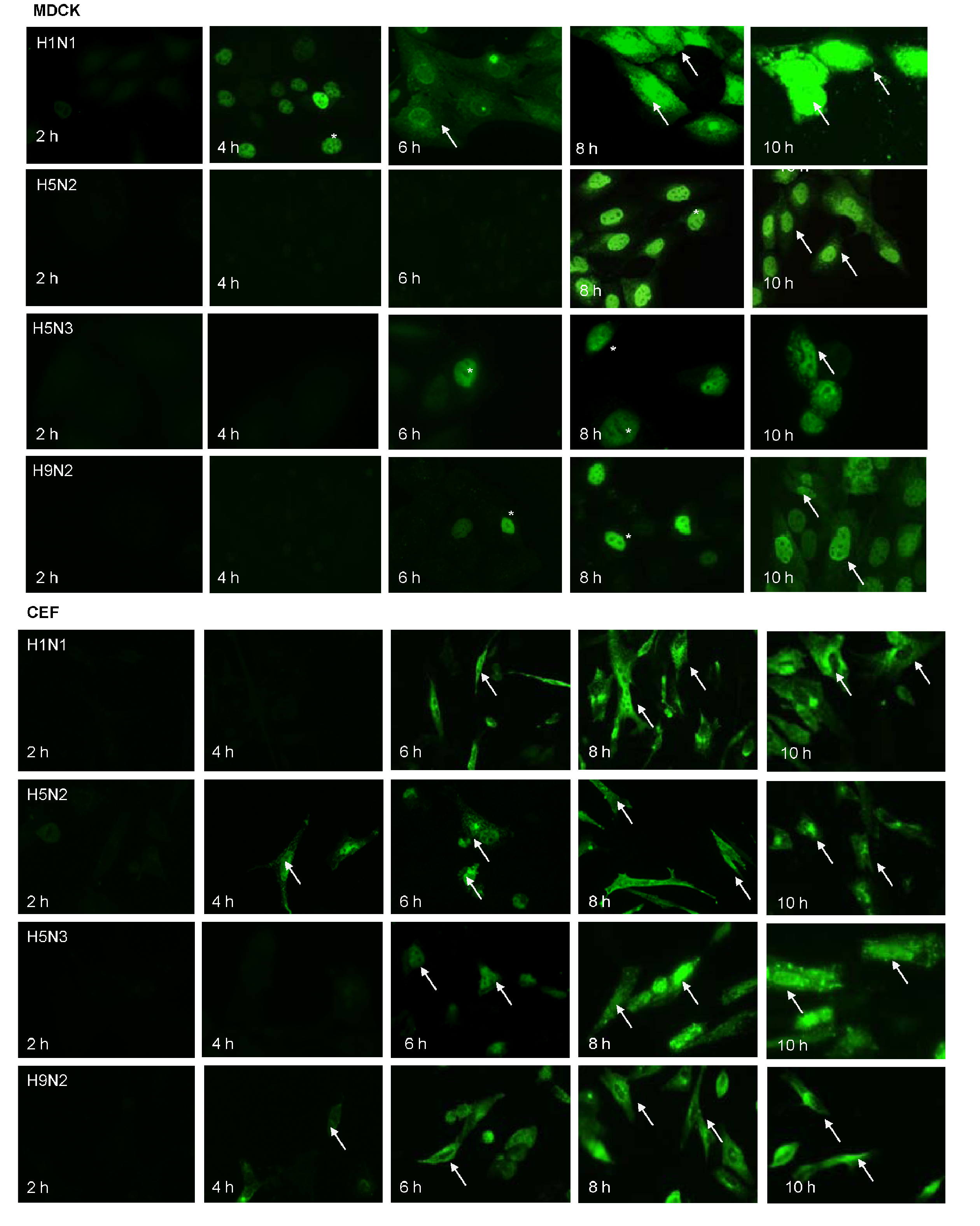

Supplement: Figure S3 — Analysis of the RNP nuclear export in AIV-infected MDCK and CEF cells. The cells were infected with either the H1N1/WSN, H9N2, H5N2/F118 or H5N3 viruses using an MOI = 4, and at specific times post infection the cells were fixed and labelled using anti-NP and goat anti-mouse conjugated to Alexa555. The stained cells were visualised using a Nikon Eclipse 80i Microscope at ×20 magnification with appropriate machine settings. The NP-stained nuclei (*) and cells (white arrow) are indicated. (TIF) [file pone.0033732.s003.tif]

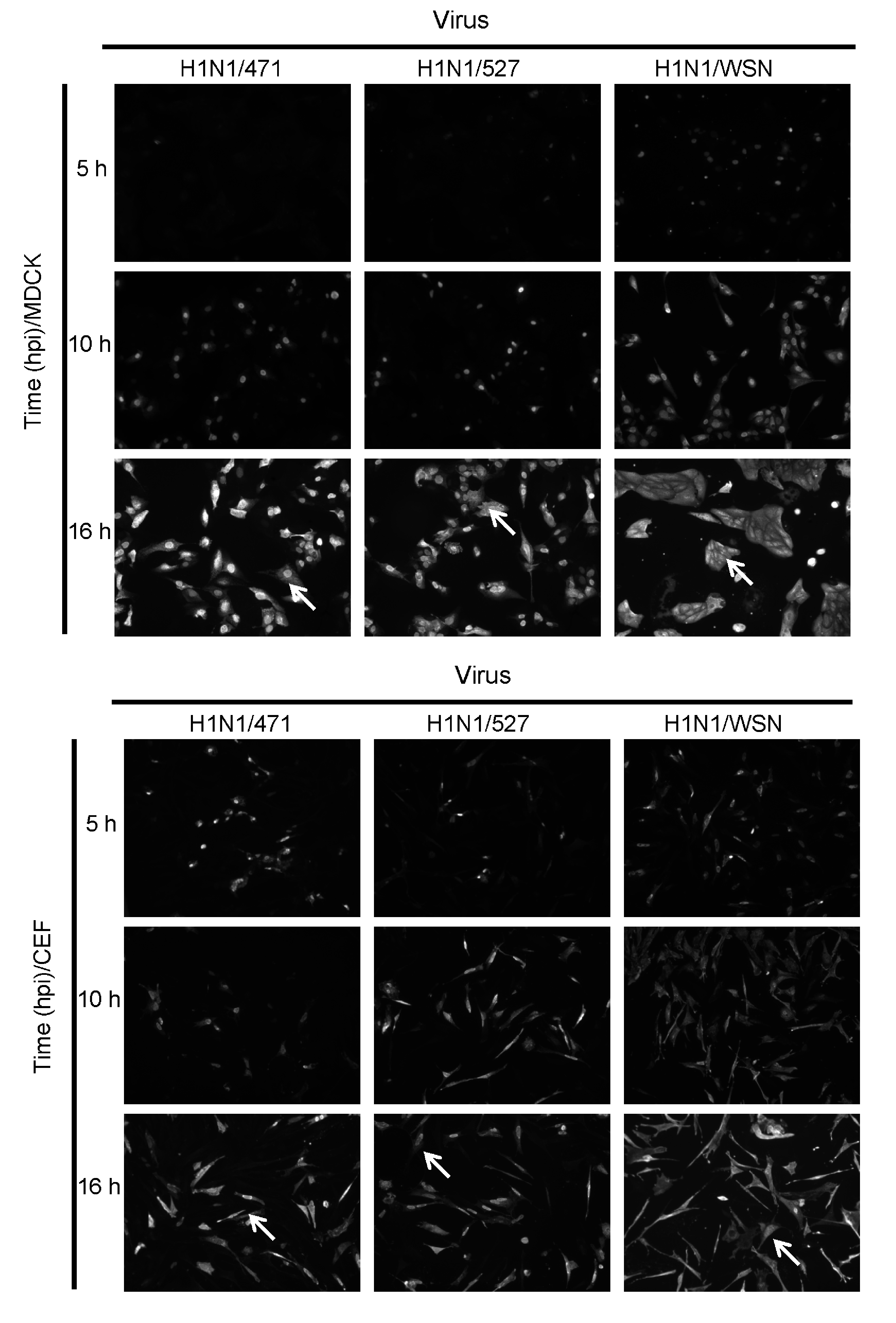

Supplement: Figure S4 — Analysis of the RNP nuclear export in pH1N1 virus-infected MDCK or CEF cells. Cells were infected with either the H1N1/WSN, pH1N1/471 or pH1N1/527 viruses using an MOI = 4. At specific times post infection the cells were fixed and labelled using anti-NP and goat anti-mouse conjugated to Alexa555. The stained cells were visualised using a Nikon Eclipse 80i Microscope at ×20 mag with appropriate machine settings. The stained cells are indicated (white arrow). (TIF) [file pone.0033732.s004.tif]

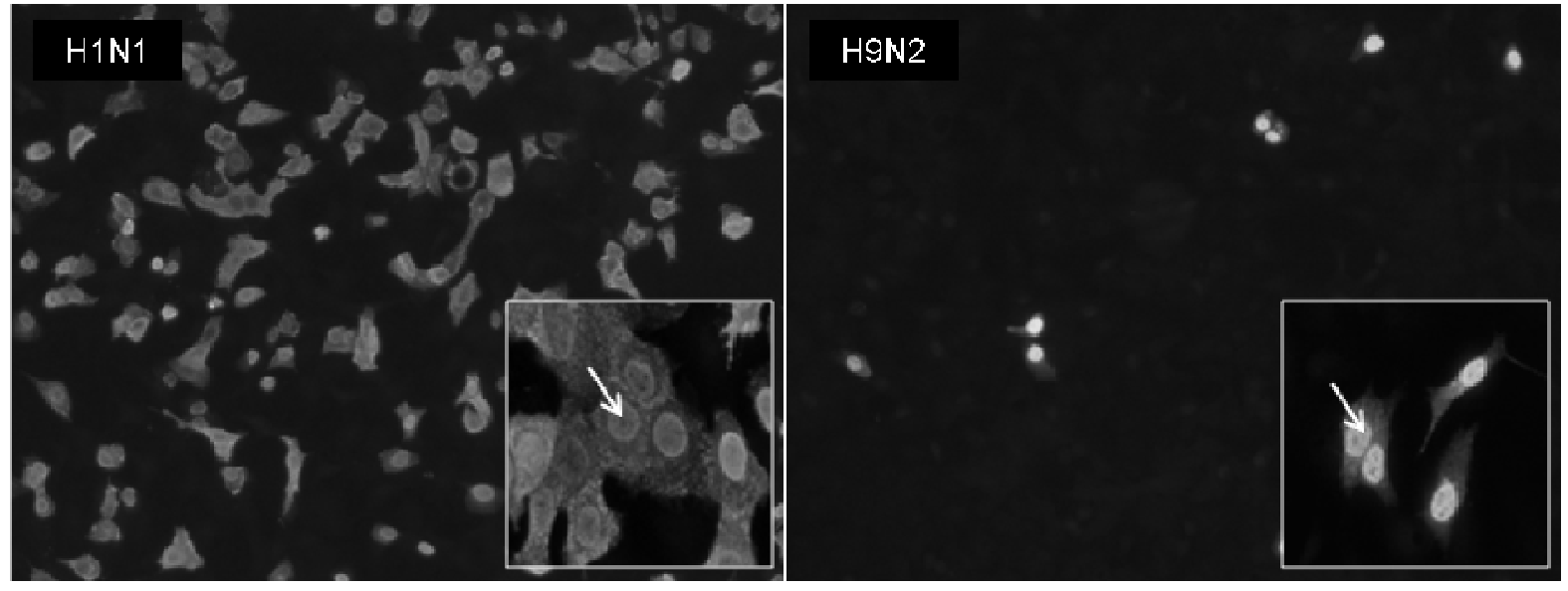

Supplement: Figure S5 — Cell-to-cell transmission in H1N1/WSN and H9N2 virus-infected A549 cell monolayers. Near confluent A549 cell monolayers were infected with either the H1N1/WSN or H9N2 viruses using an MOI = 0.01 in DMEM containing 1 µg/ml TPCK trypsin and 0.21% BSA at 37°C. At 24 hpi the cells were fixed and labelled using anti-NP and anti-mouse IgG conjugated to FITC. The labelled cells were viewed using a fluorescence microscope and the nuclei are highlighted (white arrow). Insets show the staining pattern at higher magnification. (TIF) [file pone.0033732.s005.tif]

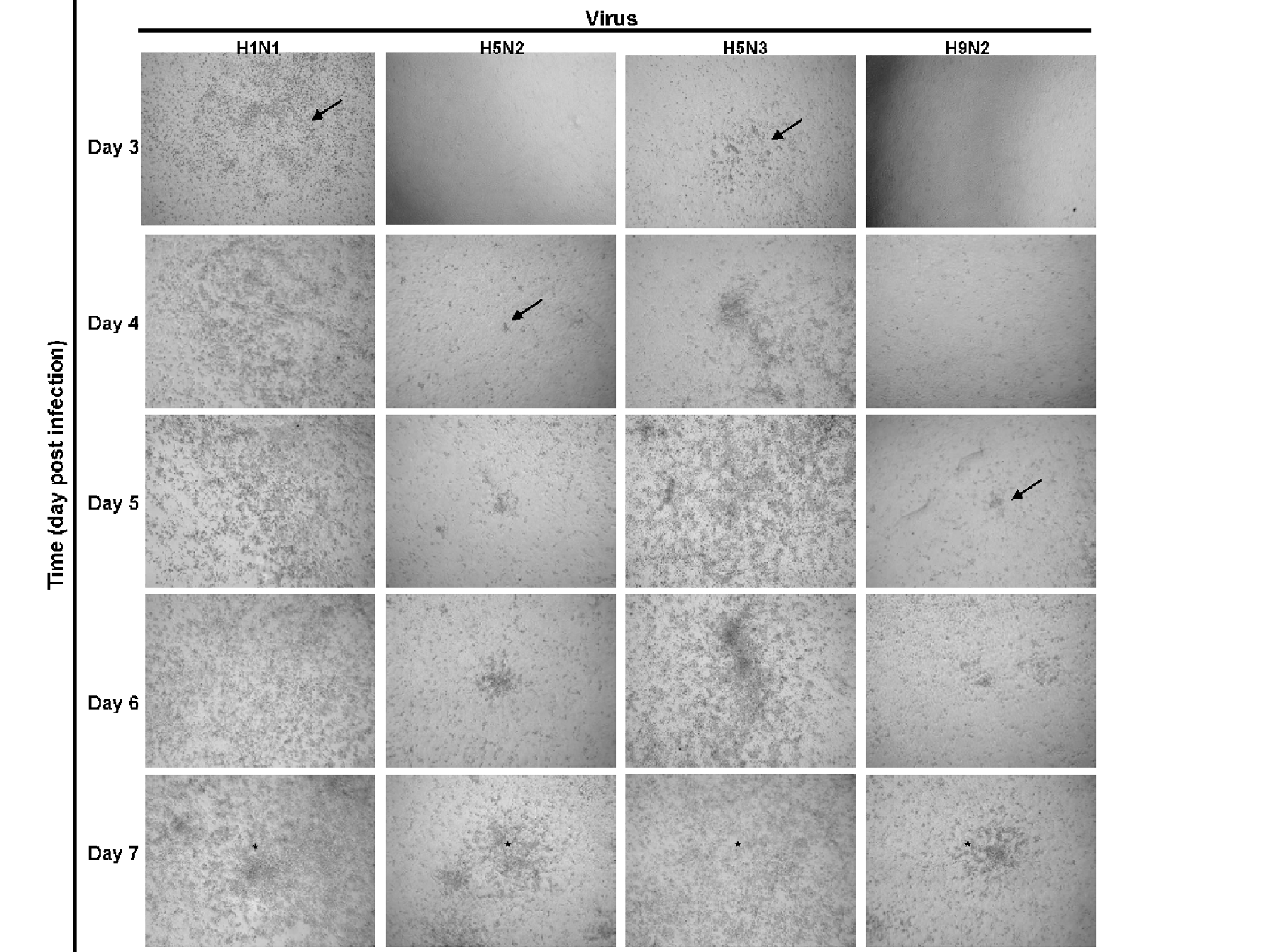

Supplement: Figure S6 — Plaque formation in MDCK cells infected with influenza virus. Near confluent MDCK cells were infected either with the H1N1/WSN, H5N2/F118, H5N3 or H9N2, and virus plaque formation monitored using an agarose overlay plaque assay over a 7 day period. The start of plaque formation (indicated by the black arrow) and the centre of the final plaque (*) imaged at 7 days post-infection are highlighted. (TIF) [file pone.0033732.s006.tif]

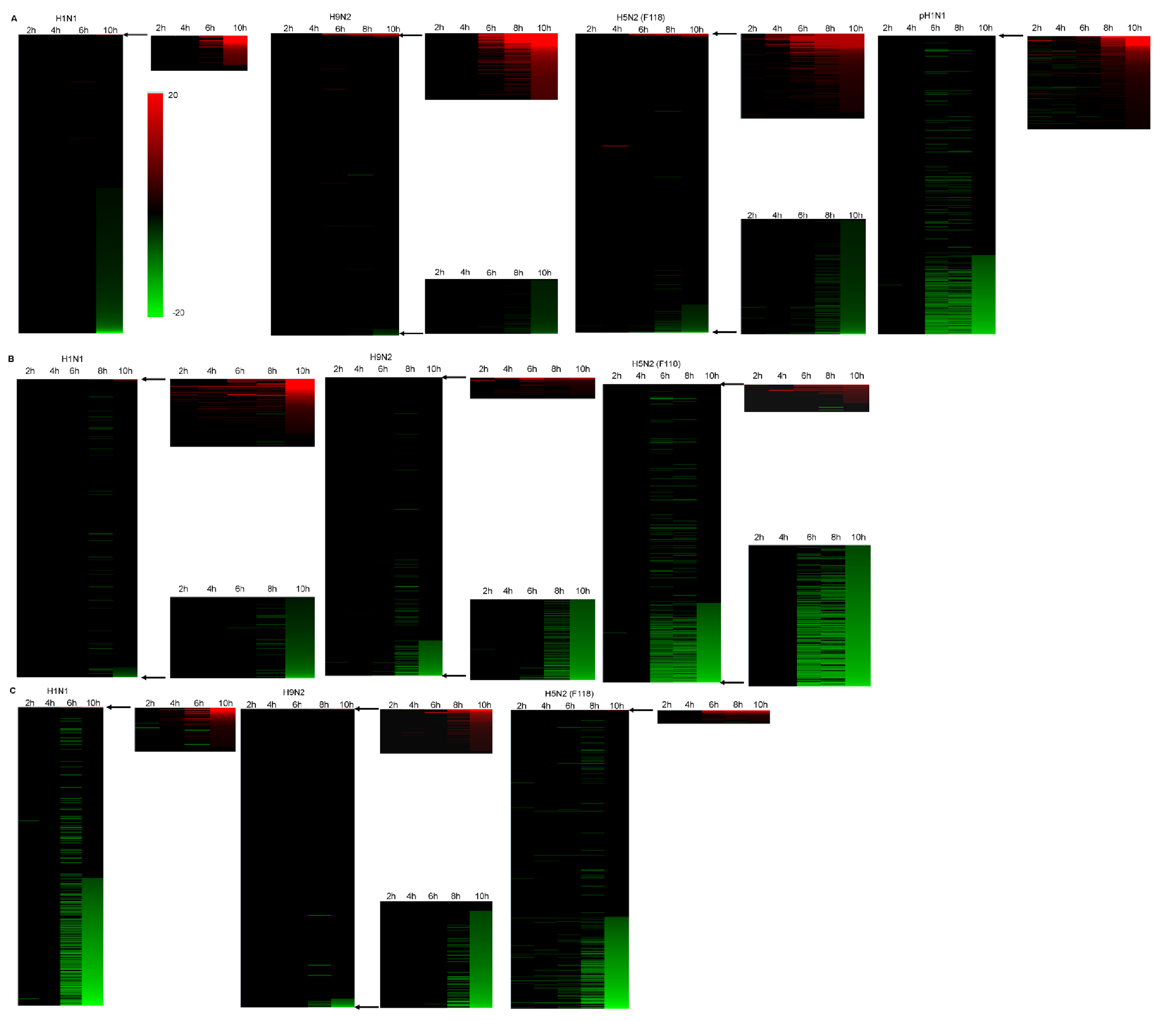

Supplement: Figure S7 — Temporal changes in the host cell transcriptome during influenza virus infection. A549, CEF and MDCK cell monolayers were either mock-infected or infected with H1N1/WSN, pH1N1/527, H5N2/F118 and H9N2 using an MOI = 4. The global host gene expression profiles in virus-infected cells were compared to mock-infected cells by microarray analysis. The fold change in gene expression in virus-infected cells is shown at each time point examined. The data were obtained from 3 independent experiments, and probe sets showing either >2 or <−2 fold change (FC) in expression are indicated (p<0.05). Expression profiles of up-regulated (red), down-regulated (green) and genes showing no change in expression (black) are shown. The insets are expanded areas showing the up-regulated and down-regulated probe sets between 2 and 10 hpi, and the colour range indicating the fold change range is also shown. (TIF) [file pone.0033732.s007.tif]

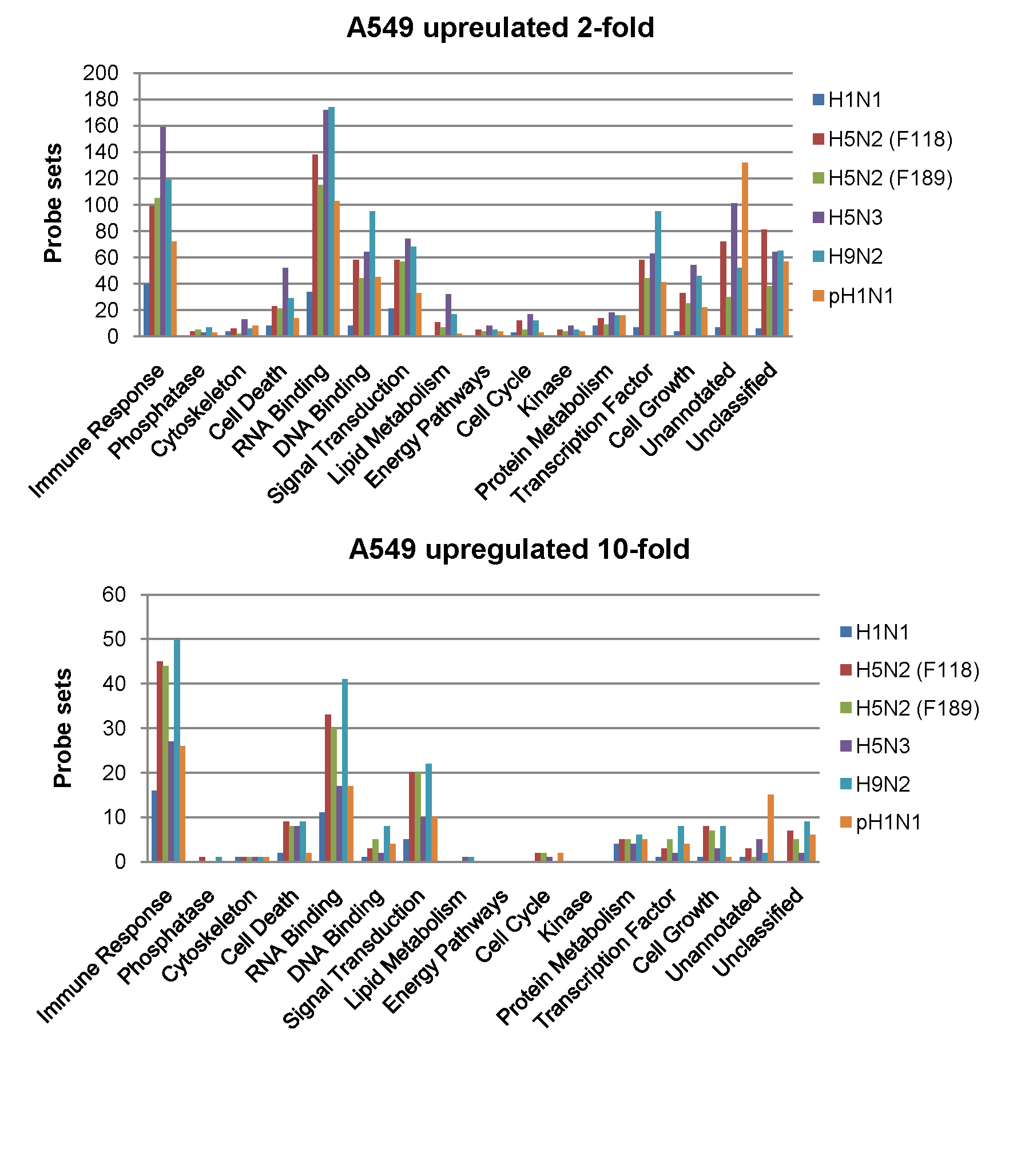

Supplement: Figure S8 — Overview of selected gene families, showing significantly up-regulated expression in A549 cells infected with influenza viruses. A549 cells were infected with either the H1N1/WSN, H9N2, H5N2/F118, H5N2/189, H5N3 or pH1N1/527 viruses at an MOI = 4 and analysed at 10 hpi. The proportion of probe sets in the different gene families, including non-annotated and unclassified gene groups, showing greater than 2-fold change in gene expression and those showing greater than 10-fold change in gene expression are presented. These are the results of 3 independent experiments, where probe sets showing either >2 or <−2 fold change (FC) in expression are indicated (p<0.05). (TIF) [file pone.0033732.s008.tif]

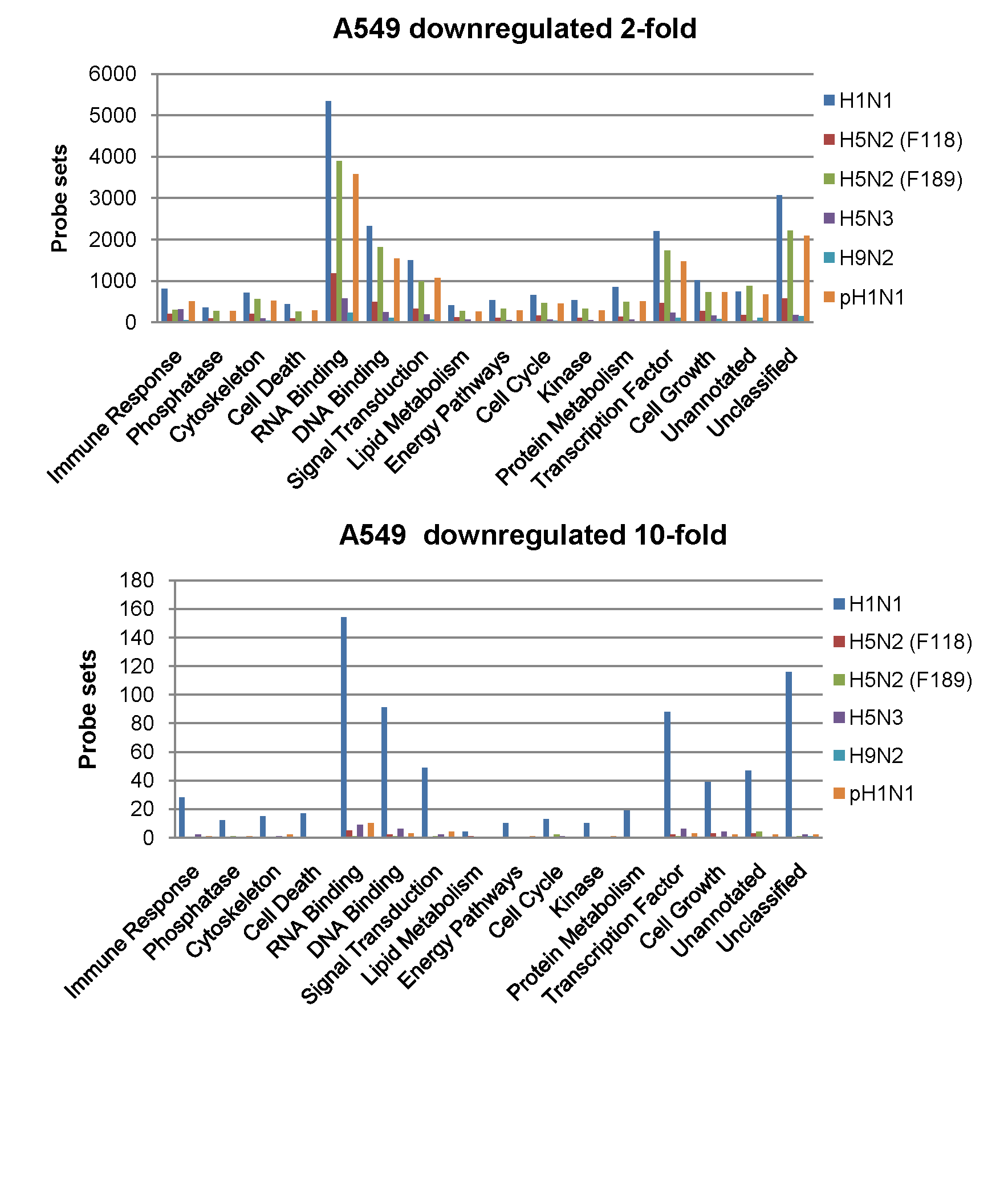

Supplement: Figure S9 — Overview of selected gene families, showing significantly down-regulated expression in A549 cells infected with influenza viruses. A549 cells were infected with either the H1N1/WSN, H9N2, H5N2/F118, H5N2/189, H5N3 or pH1N1/527 viruses at MOI = 4 and at 10 hpi, were analysed. The proportion of probe sets in the different gene families, including non-annotated and unclassified gene groups, showing less than −2-fold change in gene expression and those showing less than −10-fold change in gene expression are presented. These are the results of 3 independent experiments, where probe sets showing either >2 or <−2 fold change (FC) in expression are indicated (p<0.05). (TIF) [file pone.0033732.s009.tif]

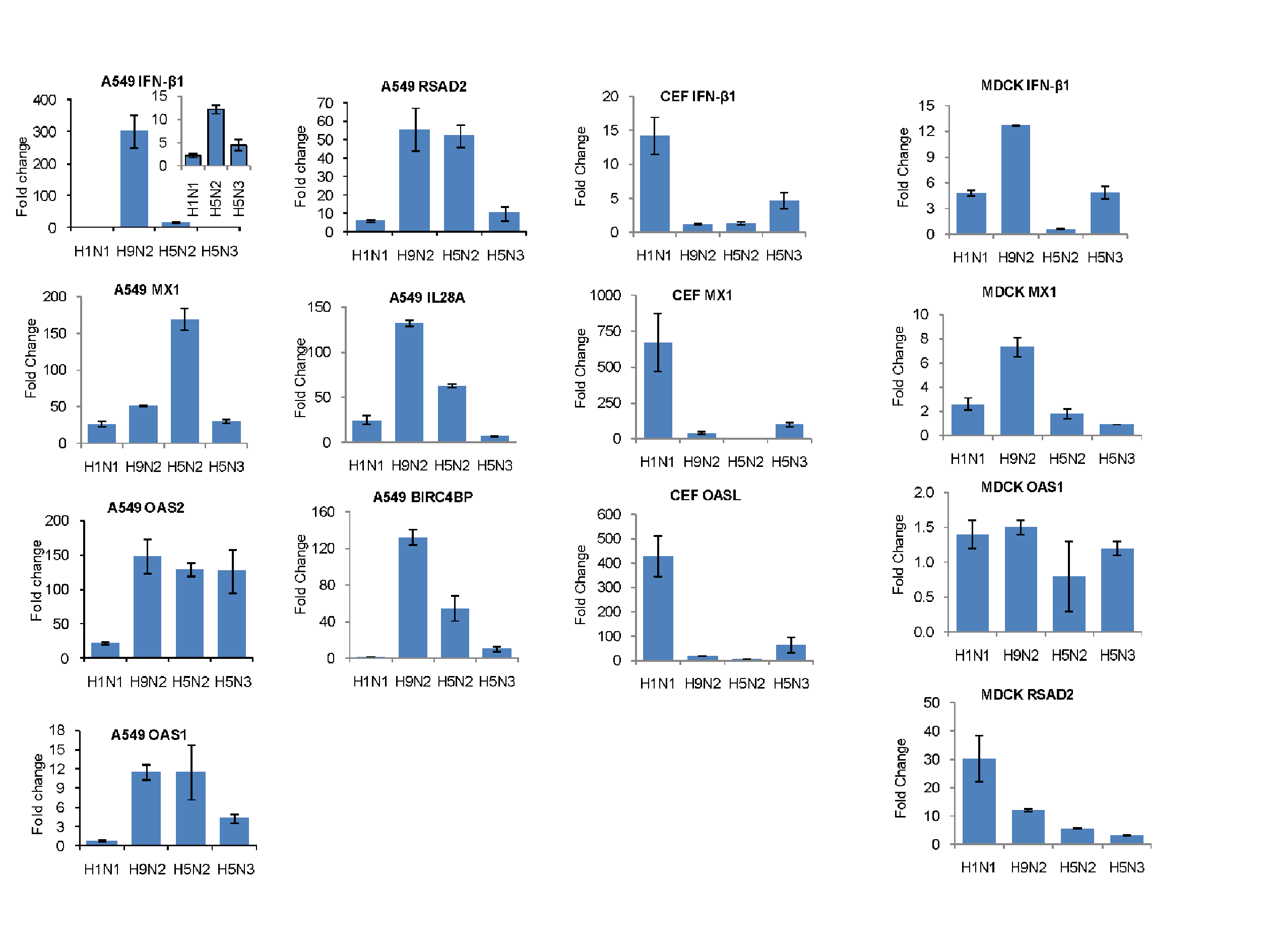

Supplement: Figure S10 — Relative expression of the selected interferon (IFN) and IFN-stimulated genes (ISGs) as measured by qPCR. A549, MDCK and CEF cells were infected with either H1N1/WSN, H9N2, H5N2/F118 or H5N3 viruses at an MOI = 4, and analysed at 10 hpi. The average values and standard error were obtained from three independent experiments (p<0.05). IFN-β1: interferon β; MX1: myxovirus resistance 1; OAS1: 2′, 5′-oligoadenylate synthase 1; OAS2: 2′, 5′-oligoadenylate synthase 2; OASL: 2′, 5′-oligoadenylate synthase-like; RSAD2: radical S-adenosyl methionine domain containing 2; IL28: interleukin 28 (interferon λ2); BIRC4BP: XIAP associated factor 1 (XAF1). (TIF) [file pone.0033732.s010.tif]

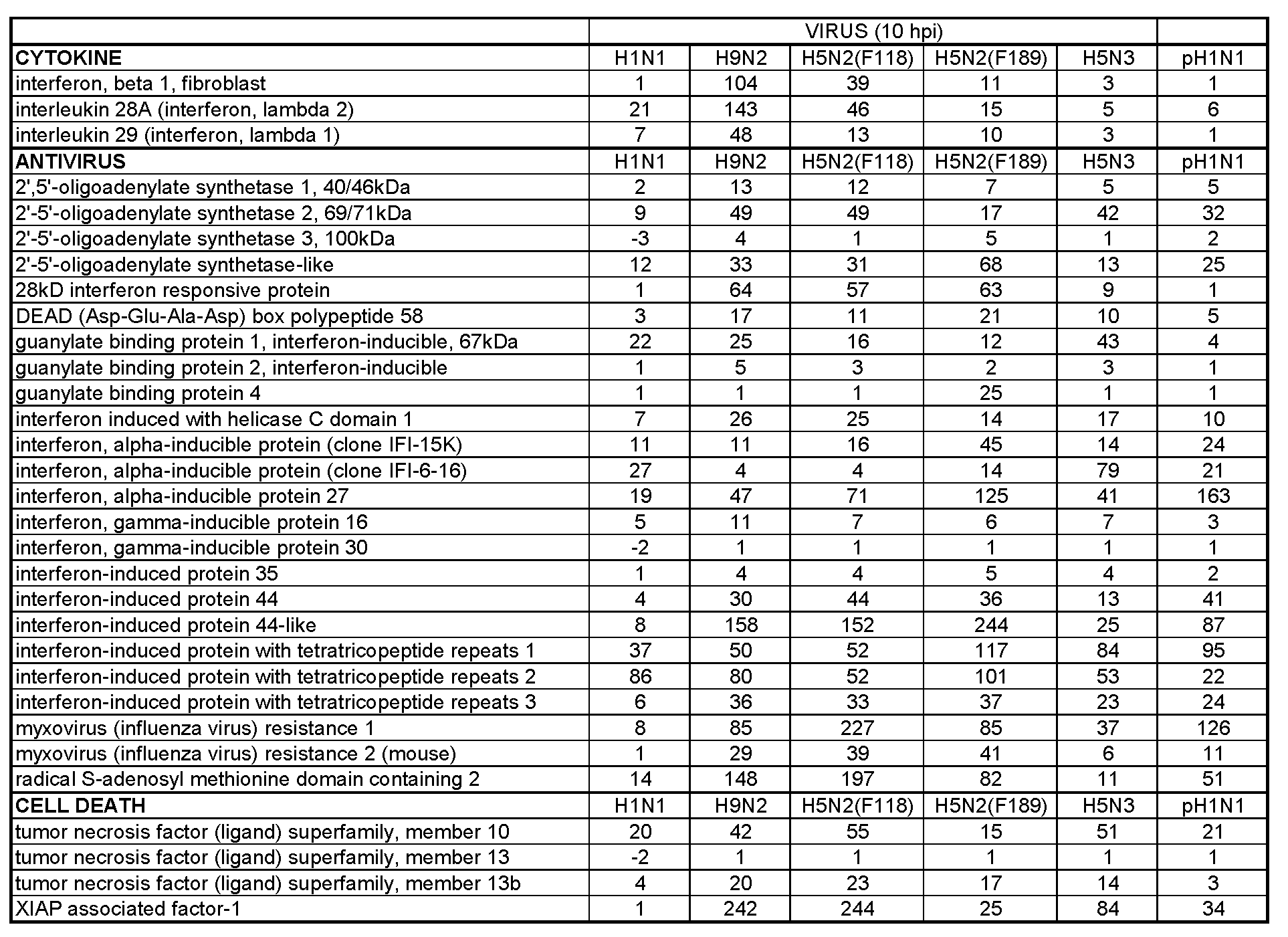

Supplement: Table S1 — Interferon, selected ISG and host genes involved in cell death pathways in influenza virus-infected A549 cells. A FC value of 1 indicates not significantly changed. These are the results of 3 independent experiments, (p<0.05). (TIF) [file pone.0033732.s011.tif]

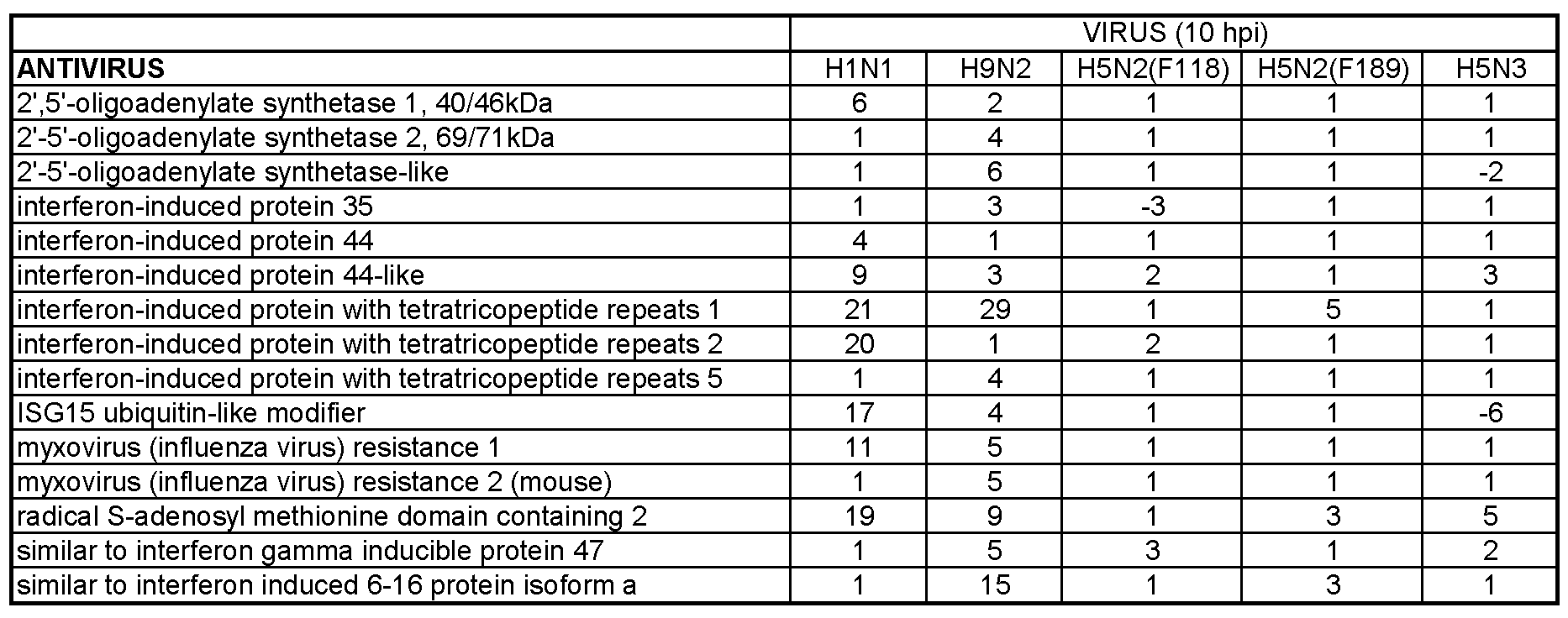

Supplement: Table S2 — Comparison of gene expression values of selected ISGs in influenza virus-infected MDCK cells. A FC value of 1 indicates not significantly changed. These are the results of 3 independent experiments, (p<0.05). (TIF) [file pone.0033732.s012.tif]

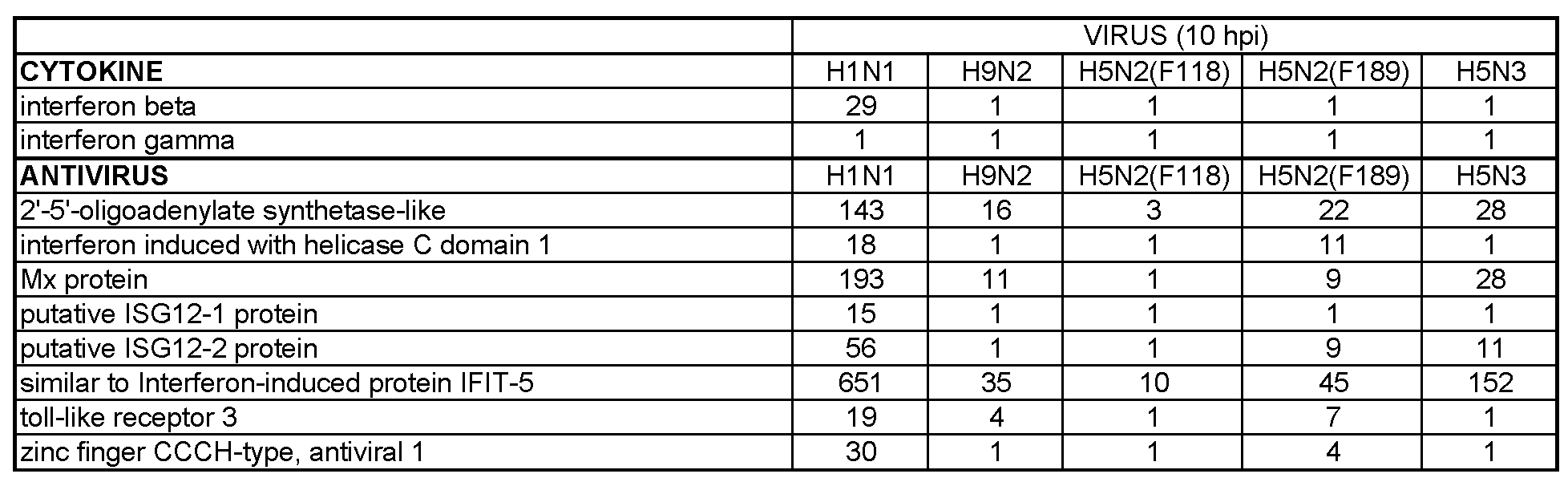

Supplement: Table S3 — Comparison of gene expression values of IFN and selected ISGs in influenza virus-infected CEF cells. A FC value of 1 indicates not significantly changed. These are the results of 3 independent experiments, (p<0.05). (TIF) [file pone.0033732.s013.tif]

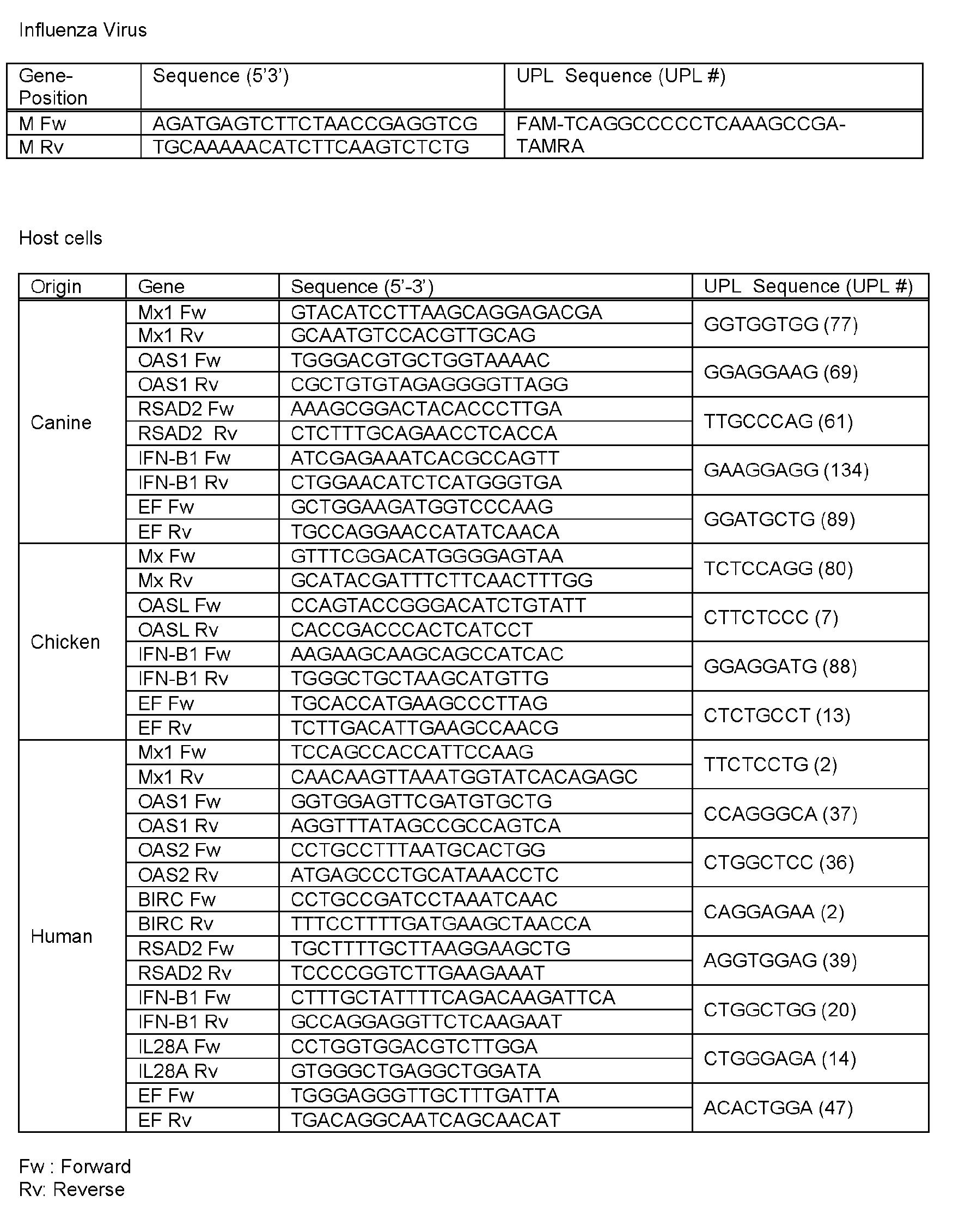

Supplement: Table S4 — Primer and probes sequences designed for real-time qPCR. Primer sequences and UPL probes (Roche) used for real-time qPCR validation of influenza M gene segment and selected canine, chicken and human host genes. IFN-β1: interferon β; MX1: myxovirus resistance 1; OAS1: 2′, 5′-oligoadenylate synthase 1; OAS2: 2′, 5′-oligoadenylate synthase 2; OASL: 2′, 5′-oligoadenylate synthase-like; RSAD2: radical S-adenosyl methionine domain containing 2; IL28: interleukin 28 (interferon λ2); BIRC4BP: XIAP associated factor 1 (XAF1); EF: elongation factor. (TIF) [file pone.0033732.s014.tif]
